# Post-marketing safety concerns with pirfenidone and nintedanib: an analysis of individual case safety reports from the FDA adverse event reporting system database and the Japanese adverse drug event report databases

**DOI:** 10.3389/fphar.2025.1530697

**Published:** 2025-04-28

**Authors:** Tao Wang, Zhiwei Cui, Yingyong Ou, Siyu Lou, Huayou Chen, Chengyu Zhu, Linmei Zhou, Fan Zou

**Affiliations:** ^1^ Department of Respiratory and Critical Care Medicine, Affiliated Hospital of Zunyi Medical University, Zunyi, China; ^2^ Department of Obstetrics and Gynecology, The First Affiliated Hospital of Xi’an Jiaotong University, Xi’an, China; ^3^ The Second Affiliated Hospital of Hainan Medical University, Haikou, Hainan, China

**Keywords:** pirfenidone, nintedanib, adverse drug events, disproportionality analysis, real-world analysis

## Abstract

**Introduction:**

To date, only two drugs, pirfenidone and nintedanib, are approved for the treatment of patients with idiopathic pulmonary fibrosis (IPF). In addition, very few studies have reported on the safety profile of either drug in large populations. This study aims to identify and compare adverse drug events (ADEs) associated with pirfenidone and nintedanib in real-world settings by analyzing data from the US Food and Drug Administration Adverse Event Reporting System (FAERS). In addition, we utilized data from the Japanese Adverse Drug Event Report (JADER) database for external validation.

**Methods:**

The ADE reports on both drugs from 2014 Q3 to 2024 Q2 in FAERS and from 2008 Q1 to 2024 Q1 in JADER were collected. After deduplication, Bayesian and non-Bayesian methods for disproportionality analysis, including Reporting Odds Ratio (ROR), Proportional Reporting Ratio (PRR), Bayesian Confidence Propagation Neural Network (BCPNN), and Multiple Gamma Poisson Shrinkers (MGPS), were used for signal detection. Additionally, time to onset (TTO) analysis were performed.

**Results:**

In total, 35,804 and 20,486 ADE reports were identified from the FAERS database for pirfenidone and nintedanib, respectively. At the system organ class (SOC) level, both drugs have a positive signal value for “gastrointestinal disorders,” “respiratory, thoracic, and mediastinal disorders,” and “metabolism and nutrition disorders.” Other positive signals for pirfenidone include “general disorders and administration site conditions,” and “skin and subcutaneous tissue disorders,” while for nintedanib, they were “investigations,” “infections and infestations,” and “hepatobiliary disorders.” Some positive signals were consistent with the drug labels, including nausea, decreased appetite, and weight decreased identified in pirfenidone, as well as diarrhea, decreased appetite, abdominal pain upper, and epistaxis identified in nintedanib. We also identified unexpected signals not listed on the drug label, such as decreased gastric pH, and pneumothorax for pirfenidone, and constipation, flatulence for nintedanib. The median onset time for ADEs was 146 days for pirfenidone and 45 days for nintedanib, respectively. Although the two antifibrotics differed in the proportion of periods in which the ADEs occurred, these ADEs were likely to continue even after a year of treatment. In the external validation of JADER, the number of reports for pirfenidone and nintedanib were 265, and 1,327, respectively. The disproportionality analysis at the SOC and preferred term (PT) levels supports the FAERS results.

**Conclusion:**

This study systematically investigates and compares the ADEs and their onset times at the SOC and specific PT levels for pirfenidone and nintedanib. Our results provide valuable pharmacological insights for the similarities and differences between the safety profiles of the two drugs and highlight the importance of monitoring and managing the toxicity profile associated with antifibrotic drugs.

## 1 Background

Idiopathic pulmonary fibrosis (IPF), the most common form of idiopathic interstitial pneumonia, is a chronic, progressive, and fatal lung disease of unknown etiology characterized by worsening respiratory symptoms and physiological impairment (Raghu et al., 2022). The incidence and prevalence of IPF vary, depending on the region, diagnostic criteria, and population. The global incidence of IPF is estimated to be 1–13 cases per 100,000 population, with a prevalence of 3–45 cases per 100,000 population (Podolanczuk et al., 2023). Unfortunately, the median survival time for patients diagnosed with IPF has been reported to be only 3 to 3.5 years (Maher, 2024). Because there is no cure for IPF, the main treatment strategy is to slow down its progression (Spagnolo et al., 2021). Despite persistent efforts, it wasn’t until antifibrotic therapy was introduced that a breakthrough occurred in the treatment of IPF. Despite this, only two drugs, pirfenidone and nintedanib, have so far been approved for the treatment of patients with IPF. These two drugs can slow down the progression of IPF by reducing the decline in lung function (King et al., 2014; Richeldi et al., 2014).

Pirfenidone is a synthetic small molecule with high oral bioavailability, significant antifibrotic activity, and excellent antioxidant and anti-inflammatory properties. In American, the recommended 2-week titration period for pirfenidone is a starting dose of 267 mg 1 tablet 3 times daily (TID) with food for 1 week, followed by 2 tablets (534 mg) TID with food for 1 week, and then 3 tablets (801 mg) TID (maintenance dose) with food (Pan et al., 2017). In Asia, on the other hand, 1800 mg pirfenidone is used as the standard dose, which is considered to be equivalent to 2,403 mg/day on a weight-standardized basis (Huang et al., 2015; Kim et al., 2023). Pirfenidone is not widely distributed in tissues; it is mainly metabolized by the cytochrome P450 (CYP) pathway, with approximately 70%–80% being metabolized by CYP1A2. Within 24 h of oral administration, approximately 80% is excreted in the urine, mainly as the major metabolite 5-carboxypyfenidone (Costabel et al., 2014; Wind et al., 2019). Several targets of pirfenidone have been proposed, including inhibition of TGF-β-related signaling pathways, inhibition of other fibrotic growth factors, e.g., platelet-derived growth factor (PDGF) and basic fibroblast growth factor (FGF), and upregulation of matrix metalloproteinases (MMPs) (Shi et al., 2011; Hisatomi et al., 2012; Hara et al., 2017). However, the antifibrotic mechanism of pirfenidone has not yet been clearly elucidated. A recent study reported that myocardin-related transcription factor signaling is a direct target of pirfenidone (Ma et al., 2023). Data from multiple clinical trials have shown that pirfenidone has a favorable benefit-risk profile. The CAPACITY program, which evaluated the safety and efficacy of pirfenidone (Noble et al., 2011), showed that in the pirfenidone 2,403 mg/day group, the most frequently reported adverse events were nausea (36%), rash (32%), dyspepsia (19%), and dizziness (18%). A phase-3 clinical trial of pirfenidone in patients with IPF (King et al., 2014) showed the most common adverse reactions were nausea (36%), rash (28.1%), headache (25.9%) and cough (70%). Gastrointestinal and skin adverse reactions were more common in the pirfenidone group, but these adverse reactions were reversible, mild, and did not leave sequelae. Additionally, several phase-2 clinical trials investigating the application of pirfenidone for other types of interstitial lung disease are currently underway. A double-blind, randomized, placebo-controlled phase 2 trial of pirfenidone in patients with unclassifiable progressive fibrosing interstitial lung disease found that the most common adverse reactions associated with pirfenidone were gastrointestinal disorders (47%), fatigue (13%) and rash (10%) (Maher et al., 2020). The most common serious adverse events in the phase 2b clinical trial of pirfenidone for the treatment of patients with progressive fibrosing interstitial lung disease other than idiopathic pulmonary fibrosis (RELIEF) were infections and infestations (8%), and adverse events such as nausea, dyspnea and diarrhoea (grade 3–4) were also observed (Behr et al., 2021). Another randomized, double-blind, placebo-controlled phase 2 study of pirfenidone in patients with rheumatoid arthritis-associated interstitial lung disease found that the most common adverse events of pirfenidone were nausea (53%), fatigue (32%), diarrhoea (31%), cough (29%) and headache (29%) (Solomon et al., 2023).

Nintedanib is a potent small-molecule inhibitor of the receptor tyrosine kinases PDGF receptor, FGF receptor, and vascular endothelial growth factor receptor (VEGFR), which are involved in fibrosis (Wollin et al., 2015). Nintedanib is approved for the treatment of IPF at a relatively fixed dose, with a recommended dose of 150 mg twice daily (Schmid et al., 2021). Nintedanib is approximately 98% bound to plasma proteins and is metabolized in the liver and intestine by hydrolytic ester cleavage to produce BIBF 1202 ZW, which is subsequently glucuronidated to form BIBF 1202 glucuronide and excreted in the feces via the biliary system. The TOMORROW study, a clinical trial on nintedanib, showed that diarrhea, nausea, and vomiting were some of the most frequently reported adverse reactions responsible for its discontinuation (Richeldi et al., 2011). The INPULSIS, a randomized, double-blind, parallel-group, and placebo-controlled clinical trial, found that nintedanib is frequently associated with diarrhea (61.5% in INPULSIS-1 and 63.2% in INPULSIS-2), nausea (22.7% in INPULSIS-1 and 26.1% in INPULSIS-2) and nasopharyngitis (12.6% in INPULSIS-1 and 14.6% in INPULSIS-2), and the majority of these reported events were mild or moderate in intensity (93.7% in INPULSIS-1 and 95.2% in INPULSIS-2) (Richeldi et al., 2014). The INPULSIS-ON study suggests that long-term use of nintedanib has a favorable safety and tolerability profile, and that diarrhea remains the most frequently reported adverse event (60.1 events per 100 patient exposure-years in patients who continued nintedanib, 71.2 events per 100 patient exposure-years in patients who initiated nintedanib) (Crestani et al., 2019). Notably, attributed to its kinase receptor inhibitory activity, the European Medicines Agency approved nintedanib in combination with docetaxel for the second-line treatment of non-small cell lung cancer patients with adenocarcinoma histology (Caglevic et al., 2015; Capelletto et al., 2019; Kim et al., 2023). A randomized phase 3 trial of nintedanib in combination with chemotherapy for non-small cell lung cancer with idiopathic pulmonary fibrosis found that the incidence of diarrhoea [68 cases (56.7%) vs. 27 cases (22.5%)] or proteinuria [56 cases (46.7%) vs. 27 cases (22.5%)] was higher in the nintedanib plus chemotherapy group compared to the chemotherapy group (Otsubo et al., 2022).

These two drugs have different mechanisms of action in the fibrosis cascade, and have different metabolic profiles, which may not only result in additive or synergistic effects but also lead to different adverse drug reactions (Huh et al., 2023). Moreover, clinical trials of these two drugs for different diseases are still ongoing, indicating their broad application prospects in the future. Although pirfenidone and nintedanib have been shown to exhibit satisfactory efficacy, there is a lack of post-marketing pharmacovigilance data systematically comparing the safety profile of these two drugs using large samples in real-world conditions. The U.S. Food and Drug Administration Adverse Event Reporting System (FAERS) and the Japanese Adverse Drug Event Report (JADER) database are two well-known system for reporting spontaneous adverse events. Through the spontaneous reporting system, the researchers can evaluate all possible associations between drugs and adverse events during post-marketing surveillance. These systems are often used in pharmacovigilance studies for drug-safety monitoring (Cui et al., 2023; Zou et al., 2023; Zou et al., 2024b). The present study conducts a pharmacovigilance analysis of pirfenidone and nintedanib using the latest data from the FAERS database and JADER database to enable and refine the safe and rational clinical use of these two drugs.

## 2 Methods

### 2.1 Data collection and deduplication

The FAERS is one of the largest publicly available ADE databases. It provides researchers with raw data from the FDA website (https://fs.fda.gov/extensions/FPDQDE-FAERS/FPD-QDE-FAERS.html). The FAERS database is updated quarterly and consists of seven datasets: demographic and administrative information (DEMO), drug information (DRUG), adverse drug reaction information (REAC), patient outcome information (OUCT), reporting source (RPSR), drug therapy start dates and end dates (THER), and indications for drug administration (INDI). In the FAERS architecture, these files were linked through specific identifiers, such as PRIMARYIDs (Shu et al., 2023b). Since both drugs were approved by the FDA in October 2014, data extraction was performed from the third quarter of 2014 (Q3 2014) to second quarter of 2024 (Q2 2024). To ensure data integrity and reliability, we strictly adhere to the official guidelines on data cleaning provided by the U.S. Food and Drug Administration, ensuring the uniqueness of the reports (Shu et al., 2022). In our study, CASEIDs (number for identifying a FAERS case), FDA_DTs (date FDA received the case), and PRIMARYIDs (unique report identifier) were used as key filters to eliminate duplicate records. We extracted the PRIMARYIDs, CASEIDs, and FDA_DTs fields from the DEMO file in the raw data and sorted them. For reports with the same CASEIDs, the most recent FDA_DT was selected. If CASEIDs and FDA_DTs were equal, the higher PRIMARYID was chosen, to remove duplicate reports submitted by different individuals and institutions (Zhu et al., 2024). Moreover, since the first quarter of 2019, each quarterly data package has included a list of deleted reports. After data deduplication, reports are excluded based on the CASEID in the deleted reports list (Shu et al., 2023a). This rigorous approach effectively eliminated redundant entries and ensured the robustness of our subsequent analyses, as each case report was assigned a unique PRIMARYID, with higher values indicating more recently submitted reports (Cao et al., 2024). The JADER database has been collecting information on cases reported by pharmaceutical companies and medical institutions since April, 2004. It consists of four main files: DEMO, DRUG, REAC, and HIST. The “DEMO” file provides essential patient information, including gender, age, and weight. The “DRUG” file contains details such as the drug’s generic name, route of administration, and the start and end dates of treatment. The “REAC” file records the name of the adverse event, its outcome, and the date it occurred. Lastly, the “HIST” file includes information regarding the patient’s underlying conditions. Data from the JADER database can be downloaded from the Pharmaceutical and Medical Devices Agency website (https://www.pmda.go.jp/index.html). Because FAERS does not use a harmonized drug coding system, we used multiple drug names for both pirfenidone (including “PIRFENIDONE,” “PIRESPA PIFENIDONE,” “'BLINDED PIRFENIDONE,” and “ESBRIET”) and nintedanib (including “NINTEDANIB,” “OFEV,” “VARGATEF,” and “BIBF 1120”) to identify ADE records associated with the administration of these two antifibrotic drugs. In JADER, “ピルフェニドン (pirfenidone)” and “ニンテダニブエタンスルホン酸塩 (nintedanib)” were used for retrieval. To improve the accuracy of the results and eliminate the potential effect of concomitant medications, we retained the role codes for adverse events only in cases where the primary suspect (PS) medication was identified for both drugs (Li et al., 2024). In FAERS, we used the system organ class (SOC) terminology to code the ADEs based on the top-level classification of the Medical Dictionary of Regulatory Activities (MedDRA, version 26.0). In JADER, adverse events were coded following the terminology recommended in MedDRA, Japanese version 26.0. We extracted all preferred terms (PTs) using MedDRA and removed those with less than three occurrences in the FAERS. After pre-processing, we screened 87,668 pirfenidone-associated PTs and 92,203 nintedanib-associated PTs in FAERS. In JADER, we screened 406 pirfenidone-associated PTs and 1,975 nintedanib-associated PTs.

### 2.2 Data analysis

**FIGURE 1:**
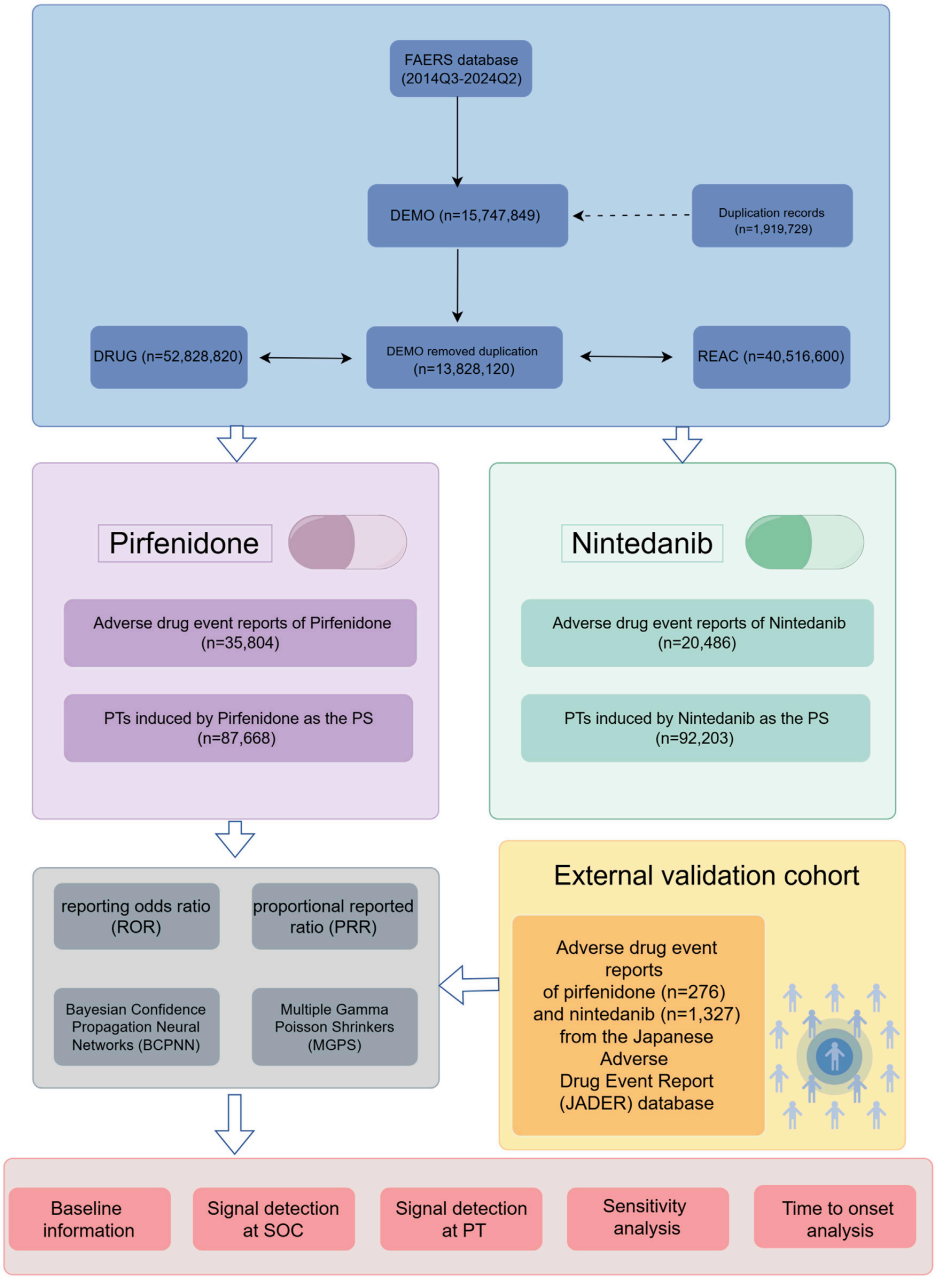
The flow chart of the study design. FAERS: Food and Drug Administration Adverse Event Reporting System; SOC, system organ class; PTs, preferred terms; PS, primary suspect.

Disproportionality analysis is an essential technique in pharmacovigilance for detecting potential links between drugs and adverse events (Montastruc et al., 2011). By comparing the actual number of reports with the expected number for each drug-adverse event combination, it helps identify signals that could suggest an elevated risk of adverse reactions, offering crucial insights for monitoring safety after a drug’s release (de Boer, 2011). The reporting odds ratios (ROR), proportional reporting ratios (PRR), Bayesian confidence propagation neural network (BCPNN), and multi-item gamma Poisson shrinker (MGPS) techniques of the disproportionality methods were used for detecting the signal strength (Jiang et al., 2024). ROR is a widely used signal detection tool in pharmacovigilance, which evaluates the potential association between a specific drug and adverse events by calculating the reporting odds ratio. This method has demonstrated high effectiveness in large-scale spontaneous reporting databases and is highly practical (Rothman et al., 2004). PRR calculates statistical indicators by comparing the risk ratio (RR) of a specific drug with the RR of the corresponding adverse reaction in the control group (Evans et al., 2001). The advantage of this method is its ability to better control the impact of different drug usage frequencies, making comparisons between drugs fairer and more accurate. However, when the denominator is zero, the calculation cannot be performed, and small denominators may cause significant fluctuations in the results (Hai et al., 2025). BCPNN is a signal detection method that combines Bayesian theory and artificial neural networks, capable of handling complex probabilistic models to address the multivariate relationships between drugs and adverse events, particularly suited for high-dimensional data analysis (Bate et al., 1998). The main advantage of BCPNN lies in its ability to quantify uncertainty, making signal detection more stable and reliable. Additionally, it excels in integrating multi-source data and cross-validation, which enhances the accuracy and credibility of the results. MGPS detects potential signals through empirical Bayesian shrinkage estimation of the reporting data, effectively reducing the occurrence of false positives. The advantage of MGPS is its ability to handle rare events and small sample sizes, providing more robust results for signal detection, especially in monitoring rare events (Dumouchel, 1999). The frequency methods (ROR and PRR) exhibit high sensitivity and are easy to compute, but the probability of false positive results increases when adverse event reports are limited. In contrast, Bayesian methods (BCPNN and MGPS) account for false positives and provide more refined results to enhance detection depth, especially for rare events. However, these methods generally have lower sensitivity, are computationally complex, and are slower in detecting signals (Li et al., 2025). Although there is no established gold standard, this study integrates four algorithms and performs cross-validation, fully leveraging the strengths of each algorithm, verifying results from multiple perspectives, and minimizing the risk of false positives to improve the detection of potential rare adverse events (Xiong et al., 2024). All the algorithms are based on a 2 × 2 list of columns (as shown in Table 1). To improve the reliability of our results, we considered only those PT terms that simultaneously satisfied all four algorithms as positive signals. We excluded ADEs related to drug indications to ensure the clarity of our statements. When conducting multiple Chi-square tests (e.g., in FAERS data analysis to examine ADE associations), each test carries a certain probability of Type I error (false-positive results). If multiple tests are performed without proper adjustment for multiple comparisons, the overall risk of Type I error increases. To reduce the risk of Type I errors, we applied the Bonferroni method to adjust the comparison of multiple *P*-values (Curtin and Schulz, 1998). The Bonferroni method is one of the simplest and most commonly used approaches for multiple comparison correction, which controls the probability of Type I error by adjusting the significance level (Zhang et al., 2023). The formula is as follows: *P*-adjusted = *P*-original×n. Where is the original *P*-value from the Chi-square test, and *P*-adjusted is the Bonferroni-corrected *P*-value. For example, in our analysis, if we examined the relationship between pirfenidone and 100 ADEs, the number of independent tests n would be 100, corresponding to 100 independent Chi-square tests. The result is considered statistically significant when *P*-adjusted < 0.05. The primary analysis of this study is presented in Figure 1.

**TABLE 1:** Methods, formulas, and thresholds for calculating Reporting Odds Ratio (ROR), Proportional Reporting Ratio (PRR), Bayesian Confidence Propagation Neural Network (BCPNN), and Empirical Bayesian Geometric Mean (EBGM).

Drug category	Target adverse drug event	Non-target adverse drug event	Sums
Target drug	a	b	a + b
Non-target drug	c	d	c + d
Total	a + c	b + d	a + b + c + d

Methods	Formula	Threshold
ROR	$ROR=\frac{a/c}{b/d}$	a≥3
	$SE\left(lnROR\right)=\sqrt{\left(\frac{1}{a}+\frac{1}{b}+\frac{1}{c}+\frac{1}{d}\right)}$	
	$95\%CI=e^{ln\left(ROR\right)\pm 1.96se}$	$95\%CI\left(lower\;limit\right)>1$
PRR	$ROR=\frac{a/\left(a+b\right)}{c/\left(c+d\right)}$	a≥3
	*χ* ^2^ = [(*ad* − *bc*)^2^](*a* + *b* + *c* + *d*)/[(*a* + *b*) (*c* + *d*) (*a* + *c*) (*b* + *d*)]	χ2≥4
BCPNN	$IC=\log_{2}\frac{p\left(x,y\right)}{p\left(x\right)p\left(y\right)}=\log_{2}\frac{a\left(a+b+c+d\right)}{\left(a+b\right)\left(a+c\right)}$	IC025>0
	$E\left(IC\right)=\log_{2}\frac{\left(a+\gamma 11\right)\left(a+b+c+d+\alpha \right)\left(a+b+c+d+\beta \right)}{\left(a+b+c+d+\gamma \right)\left(a+b+\alpha 1\right)\left(a+c+\beta 1\right)}$	
	$V\left(IC\right)=\frac{1}{{\left(ln2\right)}^{2}}\left[\frac{\left(a+b+c+d\right)-a+\gamma -\gamma 11}{\left(a+\gamma 11\right)\left(1+a+b+c+d+\gamma \right)}+\frac{\left(a+b+c+d\right)-\left(a+b\right)+a-\alpha 1}{\left(a+b+\alpha 1\right)\left(1+a+b+c+d+\alpha \right)}+\frac{\left(a+b+c+d+\alpha \right)-\left(a+c\right)+\beta -\beta 1}{\left(a+b+\beta 1\right)\left(1+a+b+c+d+\beta \right)}\right]$	
	$\gamma =\gamma 11\frac{\left(a+b+c+d+\alpha \right)\left(a+b+c+d+\beta \right)}{\left(a+b+\alpha 1\right)\left(a+c+\beta 1\right)}$	
	$IC-2SD=E\left(IC\right)-2\sqrt{V\left(IC\right)}$	
EBGM	$EBGM=\frac{a\left(a+b+c+d\right)}{\left(a+c\right)\left(a+b\right)}$	EBGM05>2
	$SE\left(InEBGM\right)=\sqrt{\left(\frac{1}{a}+\frac{1}{b}+\frac{1}{c}+\frac{1}{d}\right)}$	
	$95\%CI=e^{ln\left(EBGM\right)\pm 1.96se}$	

Variable “a” denotes the number of individuals who experience target adverse events after exposure to target drug, variable “b” represents the number of individuals who experience non-target adverse event following target drug exposure, variable “c” indicates the number of individuals experiencing target adverse event after exposure to non-target drug, and variable “d” refers to the number of individuals experiencing non-target adverse event following non-target drug exposure. 95% CI, 95% confidence interval; N, number of reports; χ2, chi-squared; IC, information component; IC025: Information Component 2.5th percentile. E (IC), IC expectations; V(IC), variance of IC; EBGM, empirical Bayesian geometric mean; EBGM05, lower limit of 95% CI of EBGM.

### 2.3 Time to onset (TTO) analysis

For this study, we defined the TTO of an ADE associated with antifibrotic drugs as the interval between the date of the onset of an ADE in the DEMO file (EVENT_DT) and the date when the administration of antifibrotic drugs was started as reported in the THER file (START_DT). In JADER, both EVENT_DT and START_DT are included in the ‘DRUG’ file. Inaccurate or missing data and the cases in which the ADE-start date was before the drug-administration date were excluded. The overall characteristics of TTOs were comprehensively evaluated using the median, quartiles, and a Weibull distribution test. Using the Weibull distribution, we can identify and estimate the increase or decrease in the incidence of ADE risk over time (Mazhar et al., 2021). The Weibull distribution test, characterized by the scale (α) and shape (β) parameters, is used to detect and predict variations in the risk of ADEs over time, with a particular emphasis on the shape parameter β in this study. When β is less than 1, and its 95% confidence interval (CI) remains below 1, it suggests a decreasing risk of adverse effects over time, indicating an early failure-type curve. On the other hand, if β is close to 1 and its 95% CI includes 1, the risk is considered stable over time, representing a random failure-type curve. Finally, if β exceeds 1 and its 95% CI does not include 1, the risk is seen as increasing over time, signifying a wear-out failure-type curve (Sauzet et al., 2013).

### 2.4 Analysis software and R packages

All data processing and statistical analyses were conducted using Microsoft Excel 2019 and R software (version 4.2.1). We utilized R packages, including “desc,” “tidyverse,” “table1,” “openxlsx,” “data.table,” “dplyr,” and “ggplot2,” for data cleaning, analysis, and visualization.

## 3 Results

### 3.1 Descriptive analysis

**FIGURE 2:**
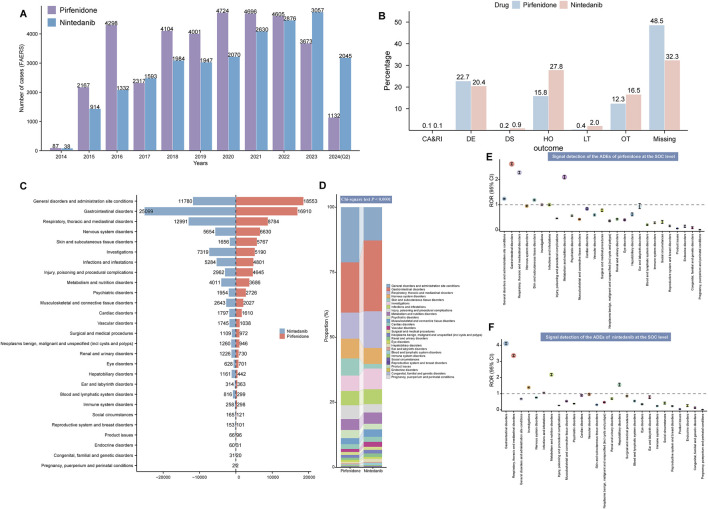
Signal detection at the SOC level. **(A)** Variation in the number of ADE reports per year since the marketing of the two antifibrotic drugs. **(B)** Overall outcome indicators of pirfenidone and nintedanib. The x-axis represents different outcome types, while the y-axis represents their proportions. **(C)** Number of ADE reports for pirfenidone and nintedanib at the SOC level. **(D)** Bar chart illustrates the difference in the composition ratio of ADE reports for the two drugs at different SOC levels. Different colors represent distinct SOC modules, and the differences in the composition ratios between the two drugs were calculated using the Chi-square test. The signal strength at the SOC level for pirfenidone **(E)** and nintedanib **(F)** is demonstrated by ROR values and their 95% CI, respectively. We set the ROR of 1 as the reference line, marked with a dashed line. When the lower limit of the ROR exceeds 1, it meets the positive threshold of the ROR algorithm and is considered a positive SOC. FAERS, Food and Drug Administration (FDA) Adverse Event Reporting System; Q2, second quarter; ADE, adverse drug event; CA, congenital anomaly; RI, require intervention; DE, death; DS, disability; HO, hospitalization; LT, life-threatening; OT, other serious outcomes; SOC, system organ class; ROR, reporting odds ratio; CI, confidence interval.

The number of ADE reports related to pirfenidone fluctuated from 2014 to 2022 but remained higher than those for nintedanib. Recent data (up to the second quarter of 2024) shows a steady increase in ADE reports for nintedanib (Figure 2A). Table 2 summarizes the clinical and demographic characteristics of pirfenidone and nintedanib from the FAERS database. A total of 35,804 and 20,486 ADE reports were identified for pirfenidone and nintedanib, respectively. The largest proportion of reports for both drugs came from patients aged 65–85 years (33.7% for pirfenidone, 49.6% for nintedanib), and males reported higher ADE rates than females (pirfenidone: 60.6% vs. 35.8%; nintedanib: 54.0% vs. 33.7%). However, more than 80% of reports lacked body weight data. The United States (86.1%) reported the highest number of ADEs for pirfenidone, followed by the United Kingdom (5.5%) and Canada (3.0%), while for nintedanib, the United States (59.3%), Japan (8.1%), and Germany (4.0%) had the most reports. Most reports for both drugs were submitted by non-health professionals [including consumer (CN), and lawyer (LW)] (pirfenidone: 68.6%, nintedanib: 56.8%). The reporting proportions of health professionals [including healthcare professional (HP), physician (MD), other health-professional (OT), and pharmacist (PH)] in the ADE reports of pirfenidone and nintedanib were 30.7% and 41.8%, respectively. The top indications for both drugs were IPF, pulmonary fibrosis, and interstitial lung disease, comprising about 70% of total cases. For pirfenidone, the most common outcomes were death (22.7%), hospitalization (15.8%), and other serious outcomes (12.3%), while for nintedanib, hospitalization (27.8%) was most frequent, followed by death (20.4%) and other serious outcomes (16.5%). Figure 2B compares the overall outcome metrics for both drugs.

**TABLE 2:** Basic information on ADEs related to pirfenidone and nintedanib from the FAERS database.

	Pirfenidone (n = 35,804)			Nintedanib (n = 20,486)	
Characteristics	Case number, n	Case proportion, %	Characteristics case number, n	Case proportion, %	
Age (years)			Age (years)		
<18	16	0.0	<18	23	0.1
18–65	2030	5.7	18–65	2,725	13.3
65–85	12056	33.7	65–85	10155	49.6
>85	1021	2.8	>85	608	3.0
Unknown	20681	57.8	Unknown	6975	34.0
Sex			Sex		
Female	12818	35.8	Female	6908	33.7
Male	21689	60.6	Male	11069	54.0
Unknown	1297	3.6	Unknown	2,509	12.2
Weight (kg)			Weight (kg)		
<50	149	0.4	<50	420	2.1
50–100	3597	10.0	50–100	2,852	13.9
>100	603	1.7	>100	232	1.1
Unknown	31455	87.9	Unknown	16982	82.9
Reported Countries (top five)			Reported Countries (top five)		
US	30826	86.1	US	12138	59.3
UK	1964	5.5	JP	1660	8.1
CA	1075	3.0	DE	825	4.0
DE	314	0.9	FR	418	2.0
FR	265	0.7	UK	394	1.9
Reported person			Reported person		
CN	24562	68.6	CN	11623	56.7
HP	3029	8.5	HP	835	4.1
LW	1	0.0	LW	3	0.0
MD	5242	14.6	MD	6146	30.0
OT	1414	3.9	OT	602	2.9
PH	1304	3.6	PH	988	4.8
Unknown	252	0.7	Unknown	289	1.4
Indications (Top five)			Indications (Top five)		
Idiopathic Pulmonary Fibrosis	25917	72.4	Idiopathic Pulmonary Fibrosis	11490	56.1
Pulmonary Fibrosis	2,565	7.2	Interstitial Lung Disease	1538	7.5
Interstitial Lung Disease	1144	3.2	Pulmonary Fibrosis	855	4.2
Pulmonary Arterial Hypertension	1180	0.5	Non-Small Cell Lung Cancer	283	1.4
Chronic Obstructive Pulmonary Disease	58	0.2	Lung Adenocarcinoma	253	1.2
Serious Outcomes			Serious Outcomes		
Other serious outcomes (OT)	4390	12.3	Other serious outcomes (OT)	3387	16.5
Hospitalization (HO)	5659	15.8	Hospitalization (HO)	5687	27.8
Death (DE)	8130	22.7	Death (DE)	4173	20.4
Life-threatening (LT)	157	0.4	Life-threatening (LT)	412	2.0
Disability (DS)	85	0.2	Disability (DS)	190	0.9
Require intervention (RI)	26	0.1	Require intervention (RI)	9	0.0
Congenital anomaly (CA)	3	0.0	Congenital anomaly (CA)	5	0.0
Missing	17354	48.5	Missing	6623	32.3

Non-health professionals include CN and LW, while health professionals include HP, MD, OT, and PH. US, United States; UK, United Kingdom; CA, Canada; DE, Germany; FR, France; JP, Japan; CN, consumer; HP, healthcare professional; LW, lawyer; MD, physician; OT, other health-professional; PH, pharmacist.

### 3.2 Signal detection at the SOC level

Table 3 shows the signal strength of ADEs associated with the two antifibrotics, categorized by SOCs. ADEs were reported across 27 organ systems for both drugs, with varying frequencies. The top three SOCs considering reported cases for both drugs were general disorders and administration site conditions, gastrointestinal disorders, and respiratory, thoracic, and mediastinal disorders, as shown in Figure 2C. A comparison of SOC composition ratios is presented in Figure 2D, with statistical analysis revealing a significant difference between the two drugs (*P* < 0.0001). Significant SOCs were identified through four disproportionality analysis methods (Table 3). Figures 2E,F show the RORs and their 95% confidence intervals for SOC signal strength. The significant SOCs for pirfenidone and nintedanib were five and six, respectively, with some overlap, including gastrointestinal disorders, respiratory, thoracic, and mediastinal disorders, and metabolism and nutrition disorders. Unique significant SOCs for pirfenidone included general disorders and administration site conditions (ROR_1_ 1.24 [95%CI 1.22–1.26]), and skin and subcutaneous tissue disorders (ROR_1_ 1.19 [95%CI 1.16–1.22]) (Figure 2E), while infections and infestation (ROR_2_ 1.05 [95%CI 1.02–1.08]), investigations (ROR_2_ 1.38 [95%CI 1.35–1.41]), and hepatobiliary disorders (ROR_2_ 1.55 [95%CI 1.47–1.65]) were significant for nintedanib (Figure 2F).

**TABLE 3:** Signal strength of ADE reports for the two antifibrotic drugs (pirfenidone and nintedanib) at the system organ class (SOC) level in the FAERS database.

SOC name	Case number_1_	Case number_2_	ROR (95% CI)_1_	ROR (95% CI)_2_	PRR (χ2)_1_	PRR (χ2)_2_	EBGM (EBGM05)_1_	EBGM (EBGM05)_2_	IC (IC025)_1_	IC (IC025)_2_
General disorders and administration site conditions	18553	11780	1.24 (1.22–1.26)	0.67 (0.66–0.69)	1.19 (658.64)	0.72 (1620.95)	1.19 (1.17)	0.72 (0.7)	0.25 (−1.42)	−0.48 (−2.15)
Gastrointestinal disorders	16910	25099	2.62 (2.58–2.67)	4.11 (4.06–4.17)	2.31 (13634.15)	3.27 (42768.44)	2.3 (2.27)	3.25 (3.21)	1.2 (−0.46)	1.7 (0.03)
Respiratory, thoracic and mediastinal disorders	8784	12991	2.28 (2.23–2.33)	3.36 (3.3–3.42)	2.15 (5634.49)	3.03 (18386.98)	2.14 (2.1)	3.01 (2.97)	1.1 (−0.57)	1.59 (−0.07)
Nervous system disorders	6630	5654	0.95 (0.92–0.97)	0.76 (0.74–0.78)	0.95 (17.44)	0.77 (414.68)	0.95 (0.93)	0.77 (0.75)	−0.07 (−1.74)	−0.37 (−2.04)
Skin and subcutaneous tissue disorders	5767	1656	1.19 (1.16–1.22)	0.31 (0.29–0.32)	1.18 (164.07)	0.32 (2516.76)	1.18 (1.15)	0.32 (0.31)	0.24 (−1.43)	−1.64 (−3.3)
Investigations	5190	7319	1 (0.98–1.03)	1.38 (1.35–1.41)	1 (0.12)	1.35 (697.1)	1 (0.98)	1.35 (1.32)	0.01 (−1.66)	0.43 (−1.24)
Infections and infestations	4801	5284	1 (0.97–1.03)	1.05 (1.02–1.08)	1 (0.04)	1.04 (10.2)	1 (0.97)	1.04 (1.02)	0 (−1.67)	0.06 (−1.6)
Injury, poisoning and procedural complications	4645	2962	0.46 (0.45–0.47)	0.27 (0.26–0.28)	0.49 (2774.72)	0.3 (5541.13)	0.49 (0.48)	0.3 (0.29)	−1.03 (−2.7)	−1.75 (−3.42)
Metabolism and nutrition disorders	3686	4011	2.1 (2.03–2.17)	2.18 (2.11–2.25)	2.06 (2030.62)	2.13 (2434.55)	2.05 (1.99)	2.12 (2.07)	1.04 (−0.63)	1.09 (−0.58)
Psychiatric disorders	2726	1954	0.57 (0.54–0.59)	0.38 (0.36–0.4)	0.58 (882.78)	0.39 (1920.81)	0.58 (0.56)	0.39 (0.38)	−0.79 (−2.45)	−1.34 (−3.01)
Musculoskeletal and connective tissue disorders	2027	2643	0.42 (0.41–0.44)	0.53 (0.51–0.55)	0.44 (1548.87)	0.54 (1077.72)	0.44 (0.42)	0.54 (0.53)	−1.19 (−2.86)	−0.88 (−2.55)
Cardiac disorders	1610	1797	0.84 (0.8–0.88)	0.89 (0.85–0.93)	0.84 (50.43)	0.89 (24.46)	0.84 (0.81)	0.89 (0.86)	−0.25 (−1.92)	−0.17 (−1.83)
Vascular disorders	1038	1745	0.6 (0.56–0.63)	0.96 (0.91–1.01)	0.6 (282.15)	0.96 (3.01)	0.6 (0.57)	0.96 (0.92)	−0.74 (−2.4)	−0.06 (−1.73)
Surgical and medical procedures	972	1109	0.78 (0.73–0.83)	0.85 (0.8–0.9)	0.78 (60.33)	0.85 (30.78)	0.78 (0.74)	0.85 (0.81)	−0.36 (−2.02)	−0.24 (−1.9)
Neoplasms benign, malignant and unspecified (incl cysts and polyps)	946	1260	0.36 (0.34–0.39)	0.46 (0.44–0.49)	0.37 (1044.47)	0.47 (781.12)	0.37 (0.35)	0.47 (0.45)	−1.43 (−3.1)	−1.09 (−2.76)
Renal and urinary disorders	730	1226	0.43 (0.4–0.46)	0.69 (0.65–0.73)	0.43 (551.08)	0.69 (170.68)	0.43 (0.41)	0.69 (0.66)	−1.2 (−2.87)	−0.53 (−2.2)
Eye disorders	701	628	0.4 (0.37–0.43)	0.34 (0.32–0.37)	0.41 (615.65)	0.35 (786.58)	0.41 (0.38)	0.35 (0.33)	−1.29 (−2.96)	−1.52 (−3.19)
Hepatobiliary disorders	442	1161	0.62 (0.56–0.68)	1.55 (1.47–1.65)	0.62 (105.41)	1.55 (224.6)	0.62 (0.57)	1.54 (1.47)	−0.69 (−2.36)	0.63 (−1.04)
Ear and labyrinth disorders	363	314	0.94 (0.85–1.05)	0.77 (0.69–0.87)	0.94 (1.23)	0.78 (20.43)	0.94 (0.87)	0.78 (0.71)	−0.08 (−1.75)	−0.37 (−2.03)
Blood and lymphatic system disorders	299	816	0.21 (0.18–0.23)	0.54 (0.5–0.58)	0.21 (913.24)	0.54 (322.13)	0.21 (0.19)	0.54 (0.51)	−2.26 (−3.93)	−0.88 (−2.55)
Immune system disorders	298	258	0.28 (0.25–0.32)	0.23 (0.21–0.26)	0.28 (541.73)	0.23 (653.02)	0.29 (0.26)	0.23 (0.21)	−1.81 (−3.48)	−2.09 (−3.76)
Social circumstances	121	165	0.32 (0.26–0.38)	0.41 (0.35–0.48)	0.32 (178.21)	0.41 (139.21)	0.32 (0.27)	0.41 (0.36)	−1.65 (−3.32)	−1.28 (−2.94)
Reproductive system and breast disorders	101	153	0.15 (0.13–0.19)	0.22 (0.19–0.26)	0.15 (474.48)	0.22 (422.34)	0.15 (0.13)	0.22 (0.19)	−2.7 (−4.37)	−2.17 (−3.84)
Product issues	96	66	0.06 (0.05–0.07)	0.04 (0.03–0.05)	0.06 (1395.76)	0.04 (1535.84)	0.06 (0.05)	0.04 (0.03)	−4.02 (−5.68)	−4.63 (−6.3)
Endocrine disorders	31	60	0.14 (0.1–0.19)	0.25 (0.19–0.32)	0.14 (170.45)	0.25 (134.88)	0.14 (0.1)	0.25 (0.2)	−2.87 (−4.54)	−2 (−3.66)
Congenital, familial and genetic disorders	20	31	0.08 (0.05–0.13)	0.12 (0.08–0.17)	0.08 (206.52)	0.12 (199.23)	0.08 (0.06)	0.12 (0.09)	−3.61 (−5.27)	−3.05 (−4.71)
Pregnancy, puerperium and perinatal conditions	2	2	0.01 (0–0.02)	0.01 (0–0.02)	0.01 (337.39)	0.01 (355.09)	0.01 (0)	0.01 (0)	−7.41 (−9.07)	−7.48 (−9.15)

Numbers 1 and 2 marked in the lower right corner refer to calculations related to pirfenidone and nintedanib, respectively. ADE, adverse drug event; ROR, Reporting Odds Ratio; PRR, Proportional Reporting Ratio; EBGM, empirical Bayesian geometric mean; EBGM05, Empirical Bayes Geometric Mean 5th percentile; IC, information component; IC025, Information Component 2.5th percentile.

### 3.3 Disproportionality analysis for both antifibrotics

**FIGURE 3:**
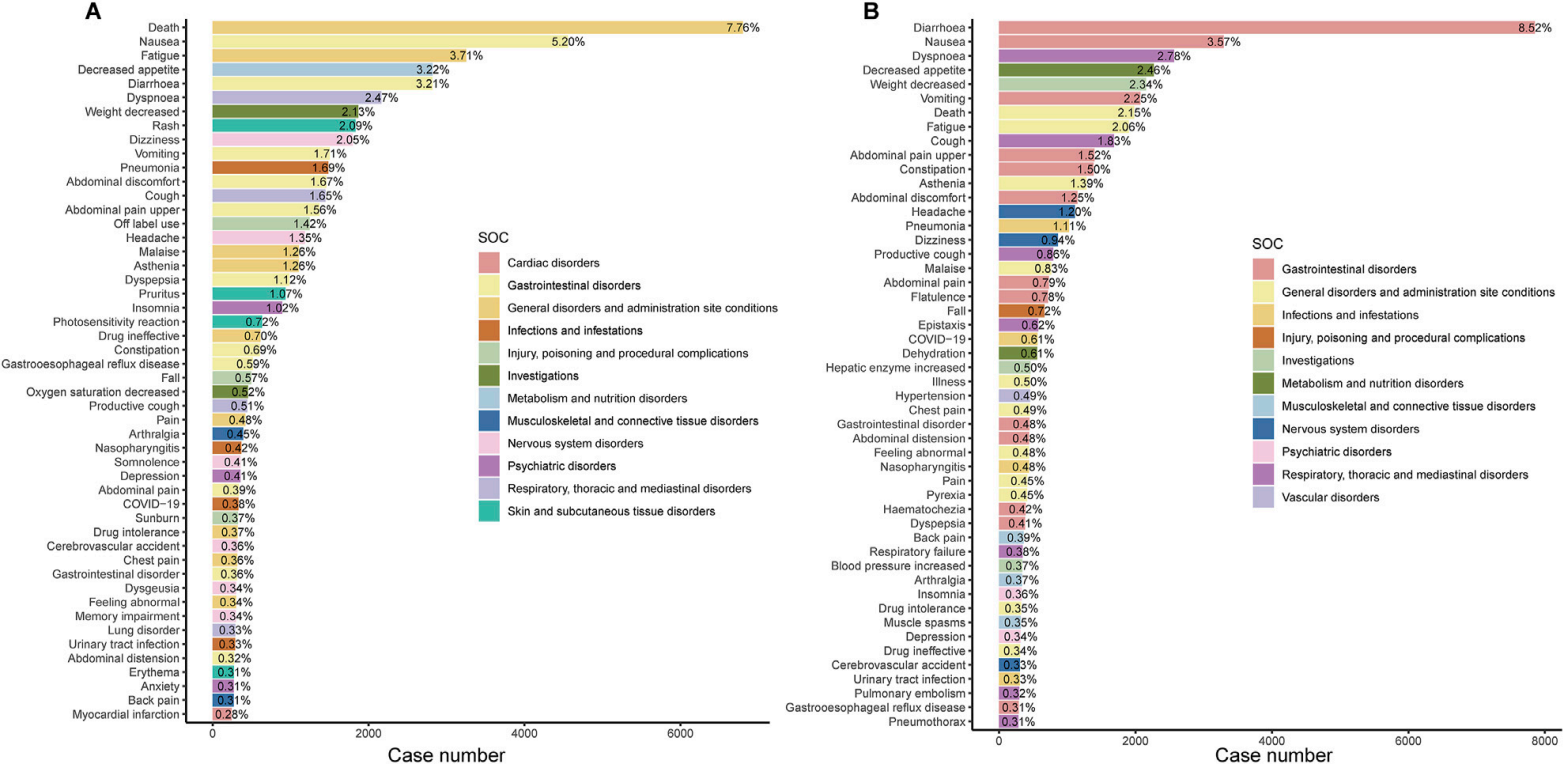
Bar chart showing the case number and frequency of the top 50 preferred terms (PTs) for pirfenidone **(A)** and nintedanib **(B)**. SOC, system organ class. The x-axis represents the number of reports for a specific PT, and the percentage is calculated as the ratio of the report count for a given PT to the total number of adverse event reports for the specified drug.

Figures 3A,B display the top 50 PT entries with the highest percentages for pirfenidone and nintedanib, categorized by case reports. For pirfenidone, the highest proportion was death (7.76%), while for nintedanib, it was diarrhea (8.52%). Pirfenidone showed higher percentages for ‘nausea’ (5.20% vs. 3.57%), ‘fatigue’ (3.71% vs. 2.06%), and ‘decreased appetite’ (3.22% vs. 2.46%), while nintedanib had higher proportions of ‘vomiting’ (2.25% vs. 1.71%), ‘weight decreased’ (2.34% vs. 2.13%), and ‘constipation’ (1.50% vs. 0.69%) (Table 4). Excluding PT as a potential indication, we identified 87 and 176 significantly disproportionate PTs for pirfenidone and nintedanib, respectively (Supplementary Tables S1, S2).

**TABLE 4:** Complete calculation results of the disproportionality analysis for the top 50 PT entries in terms of number of cases for pirfenidone and nintedanib.

SOC name	PT	Case number	ROR (95%CI)	PRR (χ2)	EBGM (EBGM05)	IC (IC025)	SOC name	PT	Case number	ROR (95%CI)	PRR (χ2)	EBGM (EBGM05)	IC (IC025)
General disorders and administration site conditions	Death	6801	5.85 (5.71-6)	5.47 (24925.44)	5.42 (5.31)	2.44 (0.77)	Gastrointestinal disorders	Diarrhoea	7854	8.47 (8.28-8.67)	7.83 (46521.75)	7.71 (7.57)	2.95 (1.28)
Gastrointestinal disorders	Nausea	4559	4.36 (4.24-4.5)	4.19 (11108.61)	4.16 (4.06)	2.06 (0.39)	Gastrointestinal disorders	Nausea	3293	2.94 (2.84-3.04)	2.87 (4035.48)	2.86 (2.78)	1.51 (-0.15)
General disorders and administration site conditions	Fatigue	3252	2.81 (2.72-2.91)	2.75 (3638.68)	2.74 (2.66)	1.45 (-0.21)	Respiratory, thoracic and mediastinal disorders	Dyspnoea	2564	3.13 (3.01-3.26)	3.07 (3587.5)	3.06 (2.96)	1.61 (-0.05)
Metabolism and nutrition disorders	Decreased appetite	2822	8.56 (8.24-8.89)	8.31 (17904.29)	8.18 (7.93)	3.03 (1.37)	Metabolism and nutrition disorders	Decreased appetite	2268	6.46 (6.2-6.74)	6.33 (10072.37)	6.25 (6.04)	2.64 (0.98)
Gastrointestinal disorders	Diarrhoea	2818	2.99 (2.88-3.1)	2.92 (3583.25)	2.91 (2.82)	1.54 (-0.12)	Investigations	Weight decreased	2153	5.19 (4.97-5.41)	5.09 (7021.63)	5.04 (4.86)	2.33 (0.67)
Respiratory, thoracic and mediastinal disorders	Dyspnoea	2164	2.77 (2.65-2.89)	2.72 (2366.69)	2.71 (2.62)	1.44 (-0.23)	Gastrointestinal disorders	Vomiting	2071	3.21 (3.08-3.36)	3.16 (3063.05)	3.15 (3.03)	1.65 (-0.01)
Investigations	Weight decreased	1867	4.71 (4.5-4.93)	4.63 (5290.59)	4.6 (4.42)	2.2 (0.53)	General disorders and administration site conditions	Death	1982	1.51 (1.45-1.58)	1.5 (338.03)	1.5 (1.45)	0.59 (-1.08)
Skin and subcutaneous tissue disorders	Rash	1833	3 (2.87-3.14)	2.96 (2380.17)	2.95 (2.83)	1.56 (-0.11)	General disorders and administration site conditions	Fatigue	1900	1.53 (1.46-1.6)	1.52 (343.21)	1.52 (1.46)	0.6 (-1.06)
Nervous system disorders	Dizziness	1796	2.65 (2.53-2.78)	2.62 (1800.33)	2.61 (2.51)	1.38 (-0.28)	Respiratory, thoracic and mediastinal disorders	Cough	1684	3.86 (3.68-4.05)	3.81 (3478.08)	3.79 (3.64)	1.92 (0.25)
Gastrointestinal disorders	Vomiting	1503	2.43 (2.31-2.56)	2.41 (1241.89)	2.4 (2.3)	1.26 (-0.4)	Respiratory, thoracic and mediastinal disorders	Idiopathic pulmonary fibrosis	1656	563.48 (523.95-606)	553.38 (403660.32)	245.18 (230.7)	7.94 (6.27)
Infections and infestations	Pneumonia	1483	3.23 (3.07-3.41)	3.2 (2234.88)	3.18 (3.05)	1.67 (0)	Gastrointestinal disorders	Abdominal pain upper	1397	4.7 (4.46-4.96)	4.64 (3964.01)	4.6 (4.4)	2.2 (0.54)
Gastrointestinal disorders	Abdominal discomfort	1460	5.68 (5.39-5.98)	5.6 (5469.72)	5.55 (5.31)	2.47 (0.81)	Gastrointestinal disorders	Constipation	1385	4.28 (4.06-4.52)	4.23 (3401.09)	4.2 (4.02)	2.07 (0.41)
Respiratory, thoracic and mediastinal disorders	Cough	1448	3.48 (3.31-3.67)	3.44 (2502.84)	3.42 (3.28)	1.78 (0.11)	General disorders and administration site conditions	Asthenia	1283	2.35 (2.22-2.48)	2.33 (971.12)	2.32 (2.21)	1.21 (-0.45)
Gastrointestinal disorders	Abdominal pain upper	1372	4.86 (4.6-5.12)	4.8 (4092.13)	4.76 (4.55)	2.25 (0.58)	Gastrointestinal disorders	Abdominal discomfort	1149	4.22 (3.98-4.47)	4.18 (2762.35)	4.15 (3.95)	2.05 (0.39)
Injury, poisoning and procedural complications	Off label use	1244	0.85 (0.8-0.9)	0.85 (33.22)	0.85 (0.81)	-0.23 (-1.9)	Nervous system disorders	Headache	1111	1.16 (1.1-1.23)	1.16 (24.78)	1.16 (1.1)	0.21 (-1.45)
Nervous system disorders	Headache	1184	1.3 (1.23-1.38)	1.3 (83)	1.3 (1.24)	0.38 (-1.29)	Infections and infestations	Pneumonia	1027	2.11 (1.99-2.25)	2.1 (592.12)	2.09 (1.99)	1.07 (-0.6)
General disorders and administration site conditions	No adverse event	1179	4.74 (4.47-5.02)	4.69 (3397.05)	4.65 (4.43)	2.22 (0.55)	Nervous system disorders	Dizziness	864	1.2 (1.12-1.28)	1.19 (27.36)	1.19 (1.13)	0.26 (-1.41)
General disorders and administration site conditions	Malaise	1106	1.66 (1.57-1.77)	1.66 (287.94)	1.65 (1.57)	0.72 (-0.94)	Respiratory, thoracic and mediastinal disorders	Productive cough	793	10.44 (9.73-11.21)	10.36 (6556.6)	10.14 (9.56)	3.34 (1.68)
General disorders and administration site conditions	Asthenia	1104	2.12 (2-2.25)	2.1 (640.63)	2.1 (2)	1.07 (-0.6)	General disorders and administration site conditions	Malaise	765	1.09 (1.01-1.17)	1.09 (5.37)	1.09 (1.02)	0.12 (-1.55)
Gastrointestinal disorders	Dyspepsia	985	7.75 (7.27-8.25)	7.67 (5629.63)	7.56 (7.17)	2.92 (1.25)	Gastrointestinal disorders	Abdominal pain	730	2.21 (2.05-2.38)	2.2 (476.29)	2.19 (2.06)	1.13 (-0.53)
Skin and subcutaneous tissue disorders	Pruritus	936	1.82 (1.7-1.94)	1.81 (339.09)	1.81 (1.71)	0.85 (-0.81)	Gastrointestinal disorders	Flatulence	718	9.11 (8.46-9.81)	9.05 (5041.6)	8.89 (8.35)	3.15 (1.49)
Psychiatric disorders	Insomnia	891	2.45 (2.29-2.61)	2.43 (749.66)	2.42 (2.29)	1.28 (-0.39)	Injury, poisoning and procedural complications	Fall	664	1.34 (1.25-1.45)	1.34 (57.99)	1.34 (1.26)	0.42 (-1.24)
Skin and subcutaneous tissue disorders	Photosensitivity reaction	634	28.85 (26.62-31.27)	28.65 (15934.59)	27.04 (25.27)	4.76 (3.09)	Respiratory, thoracic and mediastinal disorders	Dyspnoea exertional	590	10.12 (9.32-10.98)	10.06 (4707.58)	9.85 (9.2)	3.3 (1.63)
General disorders and administration site conditions	Drug ineffective	615	0.3 (0.27-0.32)	0.3 (1023.13)	0.3 (0.28)	-1.73 (-3.4)	Respiratory, thoracic and mediastinal disorders	Epistaxis	574	5.03 (4.63-5.46)	5.01 (1821.2)	4.96 (4.63)	2.31 (0.64)
Gastrointestinal disorders	Constipation	605	1.94 (1.79-2.1)	1.94 (273.19)	1.93 (1.81)	0.95 (-0.72)	Investigations	Oxygen saturation decreased	567	6.64 (6.11-7.21)	6.6 (2657.04)	6.52 (6.08)	2.7 (1.04)
Gastrointestinal disorders	Gastrooesophageal reflux disease	516	4.94 (4.52-5.38)	4.91 (1593.03)	4.87 (4.53)	2.28 (0.62)	Infections and infestations	COVID-19	564	1.51 (1.39-1.64)	1.51 (95.95)	1.5 (1.4)	0.59 (-1.08)
Injury, poisoning and procedural complications	Fall	496	1.05 (0.96-1.15)	1.05 (1.36)	1.05 (0.98)	0.08 (-1.59)	Metabolism and nutrition disorders	Dehydration	560	3.13 (2.88-3.4)	3.12 (801.62)	3.1 (2.89)	1.63 (-0.03)
Injury, poisoning and procedural complications	Intentional product use issue	468	2.74 (2.5-3)	2.73 (510.26)	2.72 (2.52)	1.44 (-0.22)	Investigations	Hepatic enzyme increased	460	4.68 (4.27-5.13)	4.66 (1310.9)	4.62 (4.28)	2.21 (0.54)
Investigations	Oxygen saturation decreased	454	5.57 (5.07-6.11)	5.54 (1671.76)	5.49 (5.08)	2.46 (0.79)	General disorders and administration site conditions	Illness	457	2.78 (2.53-3.04)	2.77 (513.19)	2.76 (2.55)	1.46 (-0.2)
Respiratory, thoracic and mediastinal disorders	Productive cough	444	6.06 (5.52-6.66)	6.04 (1844)	5.97 (5.52)	2.58 (0.91)	Vascular disorders	Hypertension	453	1.48 (1.35-1.62)	1.47 (69.2)	1.47 (1.36)	0.56 (-1.11)
General disorders and administration site conditions	Pain	419	0.44 (0.4-0.48)	0.44 (297.37)	0.44 (0.41)	-1.17 (-2.84)	General disorders and administration site conditions	Chest pain	451	1.86 (1.7-2.04)	1.86 (178.79)	1.86 (1.72)	0.89 (-0.77)
Musculoskeletal and connective tissue disorders	Arthralgia	392	0.63 (0.57-0.69)	0.63 (86.7)	0.63 (0.58)	-0.67 (-2.34)	Gastrointestinal disorders	Gastrointestinal disorder	445	3.42 (3.12-3.76)	3.41 (753.78)	3.39 (3.14)	1.76 (0.1)
Infections and infestations	Nasopharyngitis	369	1.29 (1.16-1.43)	1.29 (23.59)	1.29 (1.18)	0.36 (-1.3)	Gastrointestinal disorders	Abdominal distension	440	2.91 (2.65-3.2)	2.9 (546.57)	2.89 (2.67)	1.53 (-0.13)
Nervous system disorders	Somnolence	357	1.29 (1.16-1.43)	1.28 (22.54)	1.28 (1.18)	0.36 (-1.31)	General disorders and administration site conditions	Feeling abnormal	439	1.18 (1.08-1.3)	1.18 (12.58)	1.18 (1.09)	0.24 (-1.42)
Psychiatric disorders	Depression	356	1.22 (1.1-1.36)	1.22 (14.56)	1.22 (1.12)	0.29 (-1.38)	Infections and infestations	Nasopharyngitis	438	1.45 (1.32-1.6)	1.45 (61.82)	1.45 (1.34)	0.54 (-1.13)
Respiratory, thoracic and mediastinal disorders	Idiopathic pulmonary fibrosis	339	59.69 (53.3-66.85)	59.46 (17261.86)	52.79 (48.01)	5.72 (4.06)	General disorders and administration site conditions	Pain	415	0.41 (0.38-0.46)	0.42 (342.44)	0.42 (0.38)	-1.26 (-2.93)
Gastrointestinal disorders	Abdominal pain	339	1.07 (0.96-1.19)	1.07 (1.6)	1.07 (0.98)	0.1 (-1.57)	General disorders and administration site conditions	Pyrexia	411	0.81 (0.74-0.9)	0.81 (17.38)	0.82 (0.75)	-0.3 (-1.96)
Infections and infestations	COVID-19	331	0.93 (0.83-1.03)	0.93 (1.83)	0.93 (0.85)	-0.11 (-1.77)	Gastrointestinal disorders	Haematochezia	389	4.46 (4.03-4.93)	4.44 (1028.36)	4.41 (4.05)	2.14 (0.47)
Injury, poisoning and procedural complications	Sunburn	326	34.07 (30.44-38.14)	33.95 (9712.05)	31.69 (28.84)	4.99 (3.32)	Gastrointestinal disorders	Dyspepsia	381	2.8 (2.53-3.1)	2.79 (436.28)	2.78 (2.56)	1.48 (-0.19)
General disorders and administration site conditions	Drug intolerance	321	1.98 (1.78-2.21)	1.98 (155.35)	1.98 (1.8)	0.98 (-0.68)	Musculoskeletal and connective tissue disorders	Back pain	361	1.02 (0.92-1.13)	1.02 (0.09)	1.02 (0.93)	0.02 (-1.64)
Nervous system disorders	Cerebrovascular accident	318	1.58 (1.42-1.76)	1.58 (67.27)	1.58 (1.44)	0.66 (-1.01)	Respiratory, thoracic and mediastinal disorders	Respiratory failure	346	3.64 (3.27-4.05)	3.63 (654.14)	3.61 (3.3)	1.85 (0.18)
General disorders and administration site conditions	Chest pain	315	1.37 (1.22-1.53)	1.36 (30.61)	1.36 (1.24)	0.45 (-1.22)	Investigations	Blood pressure increased	339	1.44 (1.3-1.6)	1.44 (45.45)	1.44 (1.32)	0.52 (-1.14)
Gastrointestinal disorders	Gastrointestinal disorder	312	2.52 (2.25-2.81)	2.51 (282.31)	2.5 (2.28)	1.32 (-0.34)	Musculoskeletal and connective tissue disorders	Arthralgia	337	0.51 (0.46-0.57)	0.51 (156.34)	0.51 (0.47)	-0.96 (-2.63)
Nervous system disorders	Dysgeusia	301	3.06 (2.73-3.42)	3.05 (412.5)	3.04 (2.76)	1.6 (-0.06)	Psychiatric disorders	Insomnia	333	0.86 (0.77-0.96)	0.86 (7.5)	0.86 (0.79)	-0.22 (-1.88)
General disorders and administration site conditions	Feeling abnormal	300	0.85 (0.76-0.95)	0.85 (7.92)	0.85 (0.77)	-0.23 (-1.9)	General disorders and administration site conditions	Drug intolerance	325	1.91 (1.71-2.13)	1.91 (139.64)	1.9 (1.74)	0.93 (-0.74)
Nervous system disorders	Memory impairment	299	1.43 (1.27-1.6)	1.43 (37.99)	1.42 (1.3)	0.51 (-1.16)	Musculoskeletal and connective tissue disorders	Muscle spasms	319	1.15 (1.03-1.29)	1.15 (6.45)	1.15 (1.05)	0.2 (-1.46)
Respiratory, thoracic and mediastinal disorders	Lung disorder	291	4.24 (3.78-4.76)	4.23 (712.82)	4.2 (3.82)	2.07 (0.41)	Psychiatric disorders	Depression	316	1.03 (0.92-1.15)	1.03 (0.32)	1.03 (0.94)	0.05 (-1.62)
Infections and infestations	Urinary tract infection	289	1.15 (1.02-1.29)	1.15 (5.51)	1.15 (1.04)	0.2 (-1.47)	General disorders and administration site conditions	Drug ineffective	309	0.14 (0.13-0.16)	0.14 (1615.2)	0.14 (0.13)	-2.8 (-4.46)
Gastrointestinal disorders	Abdominal distension	278	1.93 (1.71-2.17)	1.93 (123.31)	1.92 (1.74)	0.94 (-0.72)	Nervous system disorders	Cerebrovascular accident	307	1.45 (1.3-1.62)	1.45 (42.55)	1.45 (1.32)	0.53 (-1.13)
Skin and subcutaneous tissue disorders	Erythema	274	0.89 (0.79-1)	0.89 (3.58)	0.89 (0.81)	-0.16 (-1.83)	Infections and infestations	Urinary tract infection	305	1.15 (1.03-1.29)	1.15 (6.11)	1.15 (1.05)	0.2 (-1.46)

**FIGURE 4:**
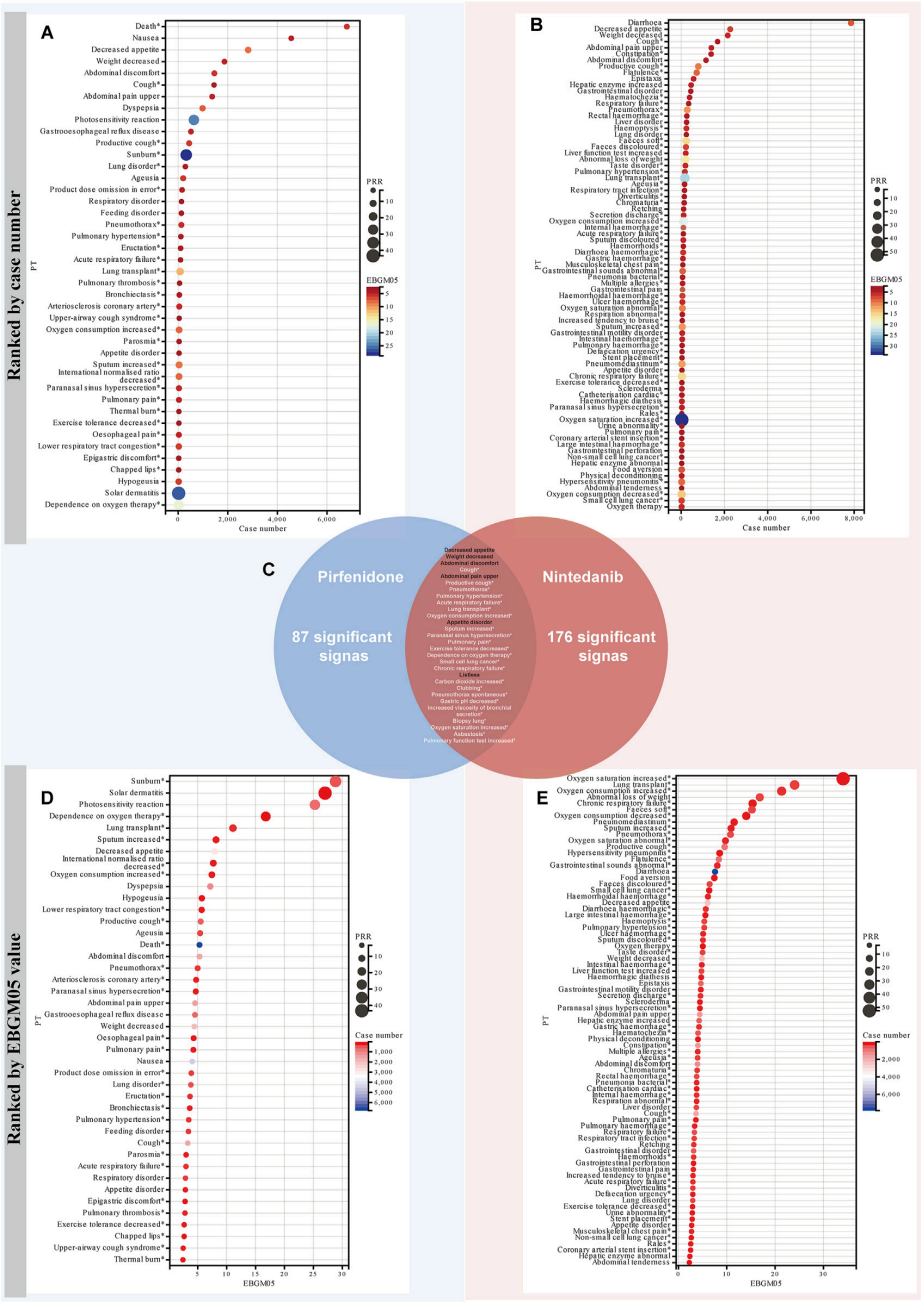
Comparison of signal strength of the ADEs for the two antifibrotic drugs at the PT level. All positive signals for pirfenidone **(A)** and nintedanib **(B)** are ranked by case number, and presented in the form of a bubble chart. The x-axis displays the number of reports, while the size and color of the circles represent the magnitude of the PRR and EBGM05 values, respectively. **(C)** Intersection of positive signals of two drugs. The Venn diagram illustrates the 29 overlapping positive signals for the two drugs. All positive signals for pirfenidone **(D)** and nintedanib **(E)** are ranked by the EBGM05 value. The x-axis displays the EBGM05 values, while the size and color of the circles represent the magnitude of the PRR values and the number of reports, respectively. The asterisk indicates that the signal is not listed on the drug label. PRR, Proportional Reporting Ratio; PT, preferred term; EBGM, empirical Bayesian geometric mean; EBGM05, the lower limit of the 95% CI of EBGM.

When categorized by case number and EBGM05 value, pirfenidone’s significant PTs included death (n = 6,801), nausea (n = 4,559), decreased appetite (n = 2,822), and weight decreased (n = 1,811) (Figure 4A). For nintedanib, significant PTs were diarrhea (n = 7,854), decreased appetite (n = 2,268), weight decreased (n = 2,153), and cough (n = 1,684) (Figure 4B). In terms of signal strength, pirfenidone had high EBGM05 values for sunburn (28.84), solar dermatitis (27.03), photosensitivity reaction (25.27), and dependence on oxygen therapy (16.77) (Figure 4D). Nintedanib had high EBGM05 values for oxygen saturation increased (34.07), lung transplant (24.03), oxygen consumption increased (21.35), and abnormal loss of weight (16.83) (Figure 4E).

Due to certain ADEs being associated with the disease, detecting these signals may confound the relationship between anti-fibrosis drugs and their ADEs (Umetsu et al., 2015). Considering that pirfenidone and nintedanib have similar indications, the analysis results of the two drugs can be used as a comparison (Xing et al., 2025). Among the two drugs, we identified a total of 29 overlapping signals (Figure 4C). Some of these overlapping signals, including unexpected positive signals, may be related to IFP progression, including pulmonary hypertension, lung transplant, chronic respiratory failure, carbon dioxide increased, pneumothorax spontaneous. However, there are also overlapping positive signals consistent with the drug labels, including decreased appetite, weight decreased, abdominal pain upper, appetite abnormal, and listlessness. Therefore, for the unexpected, overlapping positive signals, their relationship with the medication should be interpreted with caution, as they may be related to disease progression.

### 3.4 Sensitivity analysis

The evolution from monotherapy to combination therapy is a common trend in the treatment of respiratory diseases, including IPF, which helps address diagnostic uncertainties and suppress pro-inflammatory and pro-fibrotic pathways (Wuyts et al., 2014). Previous studies have indicated that the antifibrotic efficacy of pirfenidone can be enhanced when used in combination with prednisolone (Rasooli et al., 2018), while the proton pump inhibitor esomeprazole also aids in mitigating pulmonary inflammation and fibrosis (Ghebremariam et al., 2015). To eliminate the potential impact of concomitant medications on the results, we conducted a sensitivity analysis by excluding reports involving the concomitant use of pirfenidone and nintedanib. After excluding reports involving concomitant medications, we identified 21,777 reports for pirfenidone and 10,584 reports for nintedanib, corresponding to 39,195 and 27,933 adverse events, respectively. Notable persistent adverse events for pirfenidone included death, nausea, decreased appetite, weight loss, photosensitivity, dyspepsia, ageusia, and sunburn. For nintedanib, they included diarrhea, weight loss, decreased appetite, constipation, flatulence, increased hepatic enzymes, and epistaxis (Supplementary Table S3). Overall, sensitivity analyses were consistent with the main findings.

### 3.5 TTO analysis of pirfenidone- and nintedanib-related ADEs from the overall and SOC levels

**FIGURE 5:**
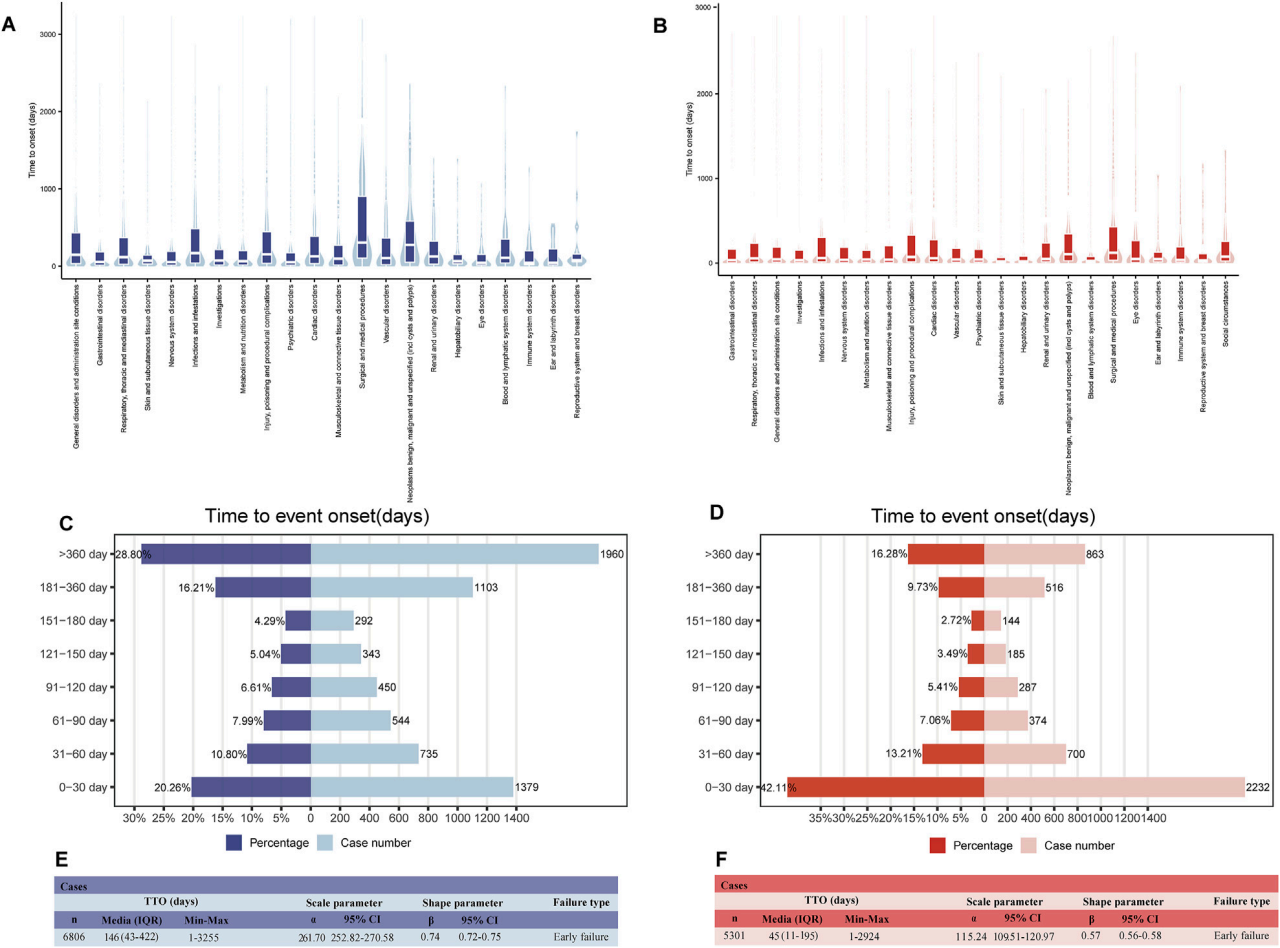
Onset time of all ADEs (counted in days). Box plot displays the time to onset (TTO) at the SOC level for pirfenidone **(A)** and nintedanib **(B)**. Bold bar within the stick: median TTO; lower end of the stick: 1/4 quantile of the TTO; upper end of the stick: 3/4 quantile of the TTO. Number and percentage of all TTO reports in different periods for pirfenidone **(C)** and nintedanib **(D)**. In the bidirectional bar chart, the y-axis displays the 8 time periods, while the right and left sides of the x-axis represent the number and proportion of TTO reports within each time period, respectively. Weibull distribution test of TTO for pirfenidone **(E)** and nintedanib **(F)**. We provided a comprehensive description of all TTO reports (median, range) as well as Weibull distribution test descriptions (scale parameter, shape parameter, and failure type). IQR, interquartile range; CI, confidence interval.

Recognizing the onset time of ADEs is crucial for better decision-making, as antifibrotic drugs can only slow IPF progression. To analyze the timing of ADEs, we examined the TTO at the SOC levels. For pirfenidone, the longest onset was 306 days [interquartile range (IQR) 106.25–905), observed in surgical and medical procedures, while immune system disorders had the shortest median onset at 43 days (IQR 17.5–200) (Figure 5A). Gastrointestinal, nervous system, psychiatric, eye, and ear and labyrinth disorders all had median onset times under two months. For nintedanib, skin and subcutaneous tissue disorders had the shortest onset at 20.5 days (IQR 7–65), and gastrointestinal, hepatobiliary, blood and lymphatic system, and reproductive system and breast had median onsets around 1 month (Figure 5B). Detailed TTOs are provided in Supplementary Table S4.

As shown in Figures 5C,D, most ADEs for pirfenidone (71.2%) occurred within the first year, while nearly half of the ADEs for nintedanib (42.1%) occurred within the first month. The median onset time for all ADEs was 146 days (IQR 43–422) for pirfenidone and 45 days (IQR 11–195) for nintedanib (Figures 5E,F). The Weibull distribution test analysis of pirfenidone showed that the shape parameter (β) was 0.74 and the upper limit of its 95% confidence interval (CI) was 0.75, whereas for nintedanib, they were 0.57 and 0.58, respectively. These values were <1, indicating a decline in the prevalence of ADEs over time (early failure type).

### 3.6 TTO analysis of pirfenidone- and nintedanib-related ADEs from PT levels

**FIGURE 6:**
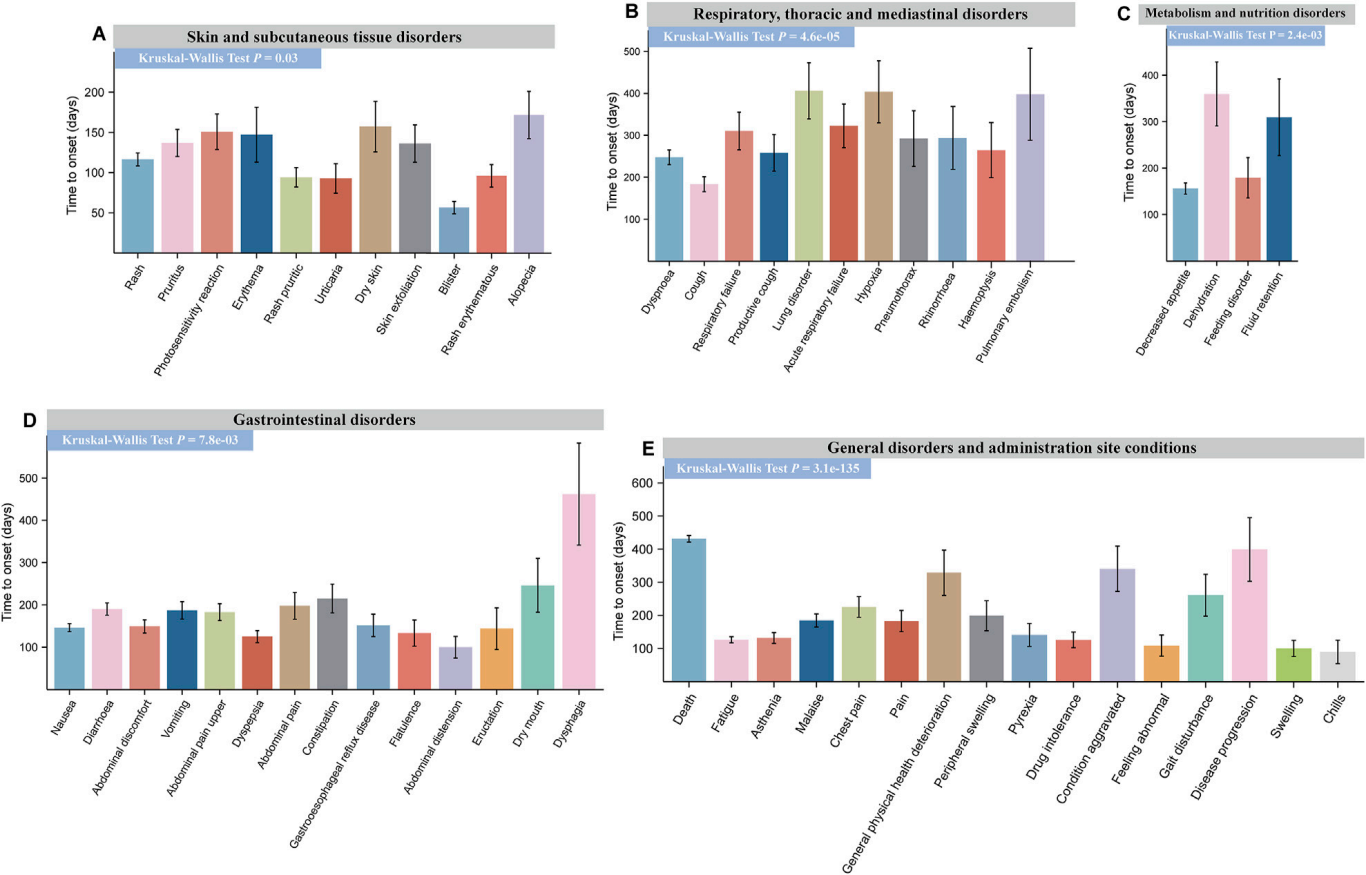
Time to onset (TTO) analysis of ADEs at the PT levels for pirfenidone. Specific comparison of TTO in PTs at six different SOC levels, including skin and subcutaneous tissue disorders **(A)**, respiratory, thoracic, and mediastinal disorders **(B)**, metabolism and nutrition disorders **(C)**, gastrointestinal disorders **(D)**, and general disorders and administration-site conditions **(E)**. The x-axis of each figure represents the PTs under the given SOC, while the y-axis represents the time of adverse event occurrence. Differences between multiple groups were calculated using the Kruskal–Wallis test. ADE, adverse drug event; PT, preferred term; SOC, system organ class.

Multiple PT terms can occur within the same SOC level. To understand variations in onset times, we compared the TTO of PTs within the same SOC. For pirfenidone, significant differences were observed in the TTO of PTs in five positive SOCs (*P* < 0.05) (Figures 6A–E). The earliest onset was for ‘abdominal distension’ in gastrointestinal disorders [96.25 days, standard deviation (SD) 164.77], while the latest was for ‘dysphagia’ (445.35 days, SD 557.22) (Figure 6D).

**FIGURE 7:**
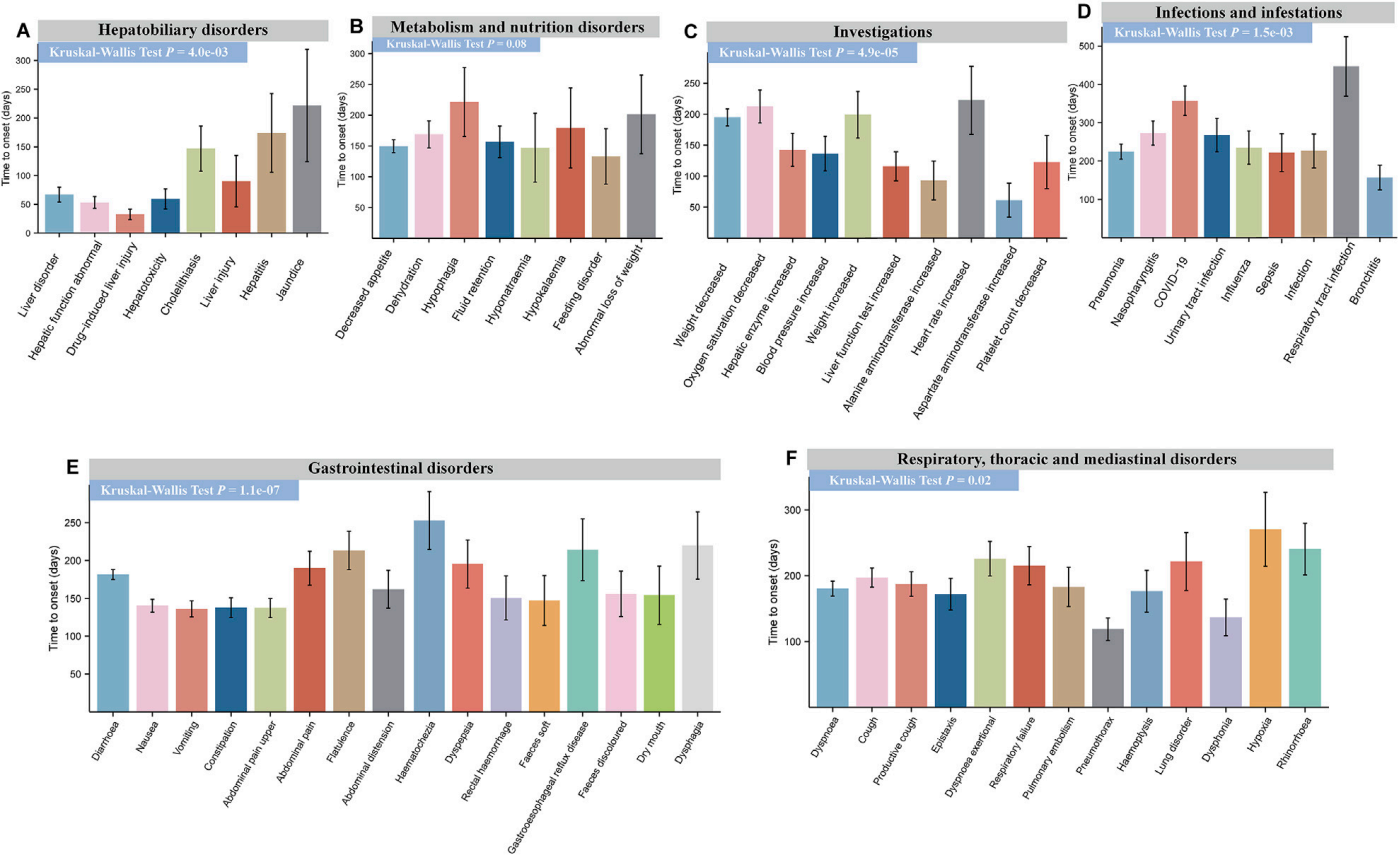
Time to onset (TTO) analysis of ADEs at the PT levels for nintedanib. Specific comparison of TTO in PTs at six different SOC levels including hepatobiliary disorders **(A)**, metabolism and nutrition disorders **(B)**, investigations **(C)**, infections and infestations **(D)**, gastrointestinal disorders **(E)**, and respiratory, thoracic, and mediastinal disorders **(F)**. The x-axis of each figure represents the PTs under the given SOC, while the y-axis represents the time of adverse event occurrence. Differences between multiple groups were calculated using the Kruskal–Wallis test. ADE, adverse drug event; PT, preferred term; SOC, system organ class.

For nintedanib, significant differences were found in TTO for PTs in hepatobiliary disorders (*P* = 4.0e-03) (Figure 7A), investigations (*P* = 4.9e-05) (Figure 7C), infections and infestations (*P* = 1.5e-03) (Figure 7D), gastrointestinal disorders (*P* = 1.1e-07) (Figure 7E), and respiratory, thoracic, and mediastinal diseases (*P* = 0.02) (Figure 7F). However, no significant difference was observed in metabolism and nutrition disorders (*P* = 0.08) (Figure 7B). The earliest PT in gastrointestinal disorder was ‘vomiting’ (136.14 days, SD 270.89), and the latest was ‘hematochezia’ (243.37 days, SD 394.39). Drug-induced liver injury occurred earliest in hepatobiliary disorders (32.55 days, SD 57.55). For more details, refer to Figures 6, 7 and Supplementary Tables S5, S6. This information helps physicians better identify and treat side effects based on when they typically occur.

### 3.7 Externally validation in JADER database

**FIGURE 8:**
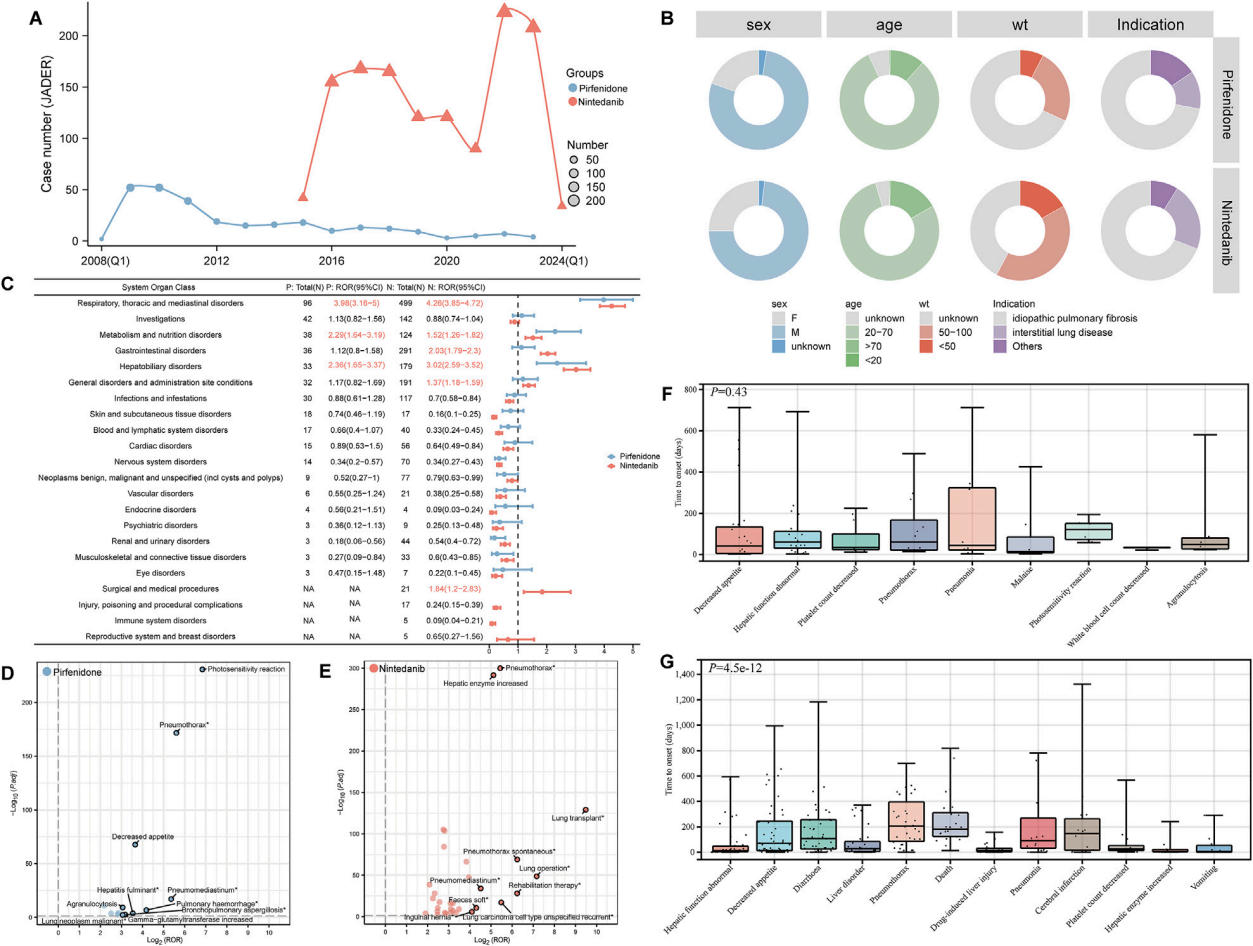
External validation was performed using the JADER database. **(A)** The annual number of ADE reports submitted for both drugs in the JADER database. **(B)** Baseline information (including sex, age, weight, and indications) for ADE reports for both drugs is displayed using a ring chart. **(C)** Signal detection at the SOC level. SOCs with a positive signal value are highlighted in red. P represents pirfenidone, and N represents nintedanib. The forest plot on the right displays the ROR values and their 95% confidence intervals. Volcano plots were created to visualize the positive risk signals for pirfenidone **(D)** and nintedanib **(E)** in JADER database. The horizontal axis represents the log2-transformed ROR values, while the vertical axis shows the -log10 of Bonferroni-adjusted *P*-values. Statistically significant signals are highlighted in color. The top 10 signals are labeled according to their log2 ROR values. The *P*-values are adjusted using the Bonferroni method. Time to onset (TTO) analysis of ADEs at the PT levels for pirfenidone **(F)** and nintedanib **(G)**. wt, weight; JADER, the Japanese Adverse Drug Event Report database; ADE, adverse drug event; SOC, system organ class; ROR, Reporting Odds Ratio; PT, preferred term.

In the external validation from JADER database, nintedanib had more reported cases than pirfenidone between 2015 and 2024. The years with the highest number of reports for pirfenidone and nintedanib were 2009 and 2022, respectively (Figure 8A). Of the 276 ADE reports for pirfenidone and 1,327 for nintedanib, baseline information was similar for both drugs (Supplementary Table S7). Males accounted for 77.9% of pirfenidone reports and 73.1% of nintedanib reports, while individuals aged 20–70 years made up 81.5% for pirfenidone and 78.9% for nintedanib. Both drugs were primarily indicated for IPF and interstitial lung disease (Figure 8B). At the SOC level, both drugs showed positive signals in respiratory, thoracic, and mediastinal disorders; metabolism and nutrition disorders; and hepatobiliary disorders (Figure 8C). Nintedanib uniquely showed positive signals in “general disorders and administration site conditions,” “gastrointestinal disorders,” and “surgical and medical procedures”. Disproportionality analysis identified 14 positive PTs for pirfenidone and 32 for nintedanib. High-case signals for pirfenidone included decreased appetite (n = 33, ROR 12.57), hepatic function abnormal (n = 21, ROR 4.54), and photosensitivity reaction (n = 11, ROR 114.56), while nintedanib had high-case signals like diarrhea (n = 101, ROR 6.97), decreased appetite (n = 84, ROR 6.83), hepatic function abnormal (n = 77, ROR 4.25), and drug-induced liver injury (n = 37, ROR 4.75), with unexpected signals for pirfenidone including pneumothorax, pneumonitis, and for nintedanib including pneumothorax, pneumatosis intestinal, and taste disorder. Volcano plots visualized significant ADE signals for both drugs, with pirfenidone showing rare events like photosensitivity reaction, pneumothorax (Figure 8D) and nintedanib showing lung transplant, lung operation signals (Figure 8E). Of the nine PTs observed, the Kruskal–Wallis test showed no significant difference in onset times for pirfenidone (*P* = 0.43), with malaise having the shortest onset (14 days) and photosensitivity the longest (121 days) (Figure 8F). For nintedanib, significant onset time differences were found among twelve PTs (*P* = 4.5e-12) (Figure 8G), with hepatic enzyme elevation having the shortest onset (7 days) and pneumothorax the longest (205.5 days).

## 4 Discussion

### 4.1 Baseline information description

Since 2018, the number of annually reported cases of pirfenidone-related ADEs has remained at a high level of over 4,000. The annual distribution of nintedanib-related ADE reports has been increasing every year since 2015. It reached a peak of 3,057 in 2023 (Figure 2A). Our results showed that ADEs related to both antifibrotics are more frequently reported in men and patients aged 65–85 years, mainly because of the higher prevalence of IPF in these populations (Raghu et al., 2016; Jo et al., 2017; Assayag et al., 2020). Possibly owing to the need for long-term use of these two antifibrotics, >55% of adverse events were reported by non-health professionals. Serious adverse outcomes, such as hospitalization and death accounted, for the majority of the two antifibrotic-related outcomes. Interestingly, among the reported countries in Table 2, pirfenidone reports were predominantly from Western countries, whereas nintedanib was reported at a relatively low rate in the United States but at a higher rate in Japan. Specifically, Asian patients appear to have a higher rate of adverse reactions compared to patients in Europe and the United States (Bonella et al., 2016; Galli et al., 2017; Toellner et al., 2017; Fletcher et al., 2018; Tzouvelekis et al., 2018; Yoon et al., 2018; Antoniou et al., 2020; Dobashi et al., 2021; Kato et al., 2021). Moreover, real-world data from Europe and the United States suggest that between 11% and 26% of patients discontinue nintedanib treatment because of ADEs (Bonella et al., 2016; Galli et al., 2017; Toellner et al., 2017; Tzouvelekis et al., 2018; Antoniou et al., 2020). However, data from Asia show that among IPF patients, this proportion is around 50% (Yoon et al., 2018; Dobashi et al., 2021; Kato et al., 2021). This difference may indeed reflect potential racial and ethnic differences in pharmacokinetics, pharmacodynamics, and overall response to these antifibrotic therapies. Racial differences in drug metabolism (e.g., variations in cytochrome P450 enzyme activity, variations in body surface area) may lead to differences in the incidence of adverse effects and discontinuation rates in different populations (Ikeda et al., 2017; Toi et al., 2019; Wells et al., 2020). liver function abnormalities and liver enzyme elevations were more frequent in Japanese patients taking nintedanib (18.4% and 15.8%, respectively) than in the overall population (2.7% and 3.3%, respectively) (Azuma et al., 2017; Hoffmann-Vold et al., 2022). Evidence, including the study by Zhao et al. suggests racial and ethnic disparities in access and response to antifibrotic therapies (Zhao et al., 2023). Black patients have lower rates of antifibrotic drug use compared with white, Hispanic, and Asian patients, and these differences can be attributed to genetic factors affecting drug metabolism, potential comorbidity differences, differences in healthcare systems and reporting methods, and cultural attitudes toward drug use (Pascoe and Smallwood, 2023; Zhao et al., 2023). In contrast, predominantly Western reports of pirfenidone may not fully reflect its safety profile in other racial or ethnic groups, and racial differences do exist in the incidence of pirfenidone adverse effects (Noble et al., 2011; King et al., 2014; Valeyre et al., 2014; Cottin et al., 2018; Chung et al., 2020; Kim et al., 2023). In addition to racial differences, other factors may also affect the reporting of drug side effects in different countries and regions, such as differences in healthcare systems, cultural differences and reporting habits, as well as the tendency of clinical trials in different regions. For example, US companies are required to submit case reports that include non-serious ADEs, but Japanese companies are not required to report known non-serious ADEs (Nomura et al., 2015). Another bibliometric analysis showed that the top three countries for the number of publications on pirfenidone were the United States (130 articles, 16.15%), China (102 articles, 12.67%) and Japan (101 articles, 12.55%), followed by nintedanib, which was published in Japan (111 articles, 14.84%), the United States (103 articles, 13.77%) and Germany (102 articles, 13.64%) (Liu J. et al., 2024). It is important to note that the differences in the incidence of adverse reactions may also be due to different therapeutic indications (Onakpoya et al., 2015). There are also a small number of reports (3%) of lung cancer as an indication for nintedanib. Therefore, these complex factors must be considered comprehensively when interpreting these differences. Understanding the ethnic and regional differences in adverse events is critical to developing global treatment guidelines and optimizing treatment regimens. Adverse events can lead to higher discontinuation rates, and in some regions more frequent monitoring and support is needed to manage side effects. Regulatory and cultural factors also have an impact, as certain populations may be more sensitive to side effects or more likely to discontinue treatment, which can affect adherence and treatment outcomes. To improve the use of medicines worldwide, clinical trials should include diverse populations to obtain representative data, and global treatment guidelines should provide tailored recommendations to optimize the use of medicines, reduce adverse reactions and improve adherence in all regions. In conclusion, recognizing these differences in baseline information can help to better understand the similarities and differences between the ADEs of the two antifibrotic drugs.

### 4.2 Signal detection at SOC

The three most common SOCs for pirfenidone and nintedanib were identical, namely “general disorders and administration-site conditions,” “gastrointestinal disorders,” and “respiratory, thoracic, and mediastinal disorders.” These results are consistent with the safety information documented in the labeling and previous clinical studies of both drugs. Furthermore, our analysis highlighted several significant and similar toxicity profiles for both antifibrotic drugs. Significant SOCs for both drugs included “gastrointestinal diseases,” “metabolic and nutritional disorders,” and “respiratory, thoracic, and mediastinal disorders.” Firstly, gastrointestinal diseases as well as metabolic and nutritional disorders are common concerns. The most common gastrointestinal and metabolic ADEs in our research, based on the percentage of numbers reported, were nausea, decreased appetite, diarrhea with pirfenidone and diarrhea, nausea, and decreased appetite with nintedanib. These results are consistent with the previous findings (Ogura et al., 2015; Galli et al., 2017; Barratt et al., 2018; Cottin et al., 2022). However, an anonymous, real-world network survey of IPF patients in the Netherlands on side effects found that nintedanib caused a significant increase in diarrhea, vomiting, and loss of appetite, while pirfenidone caused a loss of appetite (Proesmans et al., 2019). Similarly, our analysis of the composition of two antifibrotics at the SOC level revealed that “gastrointestinal disorders” accounted for a higher proportion of side effects resulting from the use of nintedanib. Nevertheless, similar levels of nausea, decreased appetite, vomiting, and abdominal discomfort have been reported with pirfenidone as with nintedanib. Hence, we should be careful about the gastrointestinal side effects of both drugs in practice and be also wary of possible additive effects. Secondly, “respiratory, thoracic, and mediastinal disorders” also accounted for a high proportion of ADEs resulting from the use of both drugs. Cough and productive cough were the two most common and significant ADEs related to “respiratory, thoracic, and mediastinal disorders” for both drugs. A clinical trial of inhaled pirfenidone solution for the treatment of IPF reported treatment-emergent adverse cough reactions (14 cases, 15.4%) (West et al., 2023). A meta-analysis on the safety of nintedanib found cough to be a common adverse drug reaction, accounting for approximately 10% of cases (Chen et al., 2021). Fortunately, the cough caused by both drugs is mild and both drugs reduce cough in the vast majority of patients (Harari et al., 2022). Thirdly, infections are another potential problem with antifibrotic therapy. In our research, the most common ADE in this SOC, measured by the percentage of numbers reported, was pneumonia for both drugs. Among the PTs at this SOC level, pneumonia (*n* = 1,483, ROR 3.23 [3.07–3.41]) was the most common ADE with pirfenidone, whereas diverticulitis (*n* = 146, ROR 3.43 [2.92–4.04]) had more reported cases in nintedanib than pirfenidone. However, the relationship between pneumonia and pirfenidone is unclear and may be related to the immunomodulation of pirfenidone (Visner et al., 2009; Bizargity et al., 2012). In addition, there is no direct evidence of a link between diverticulitis and nintedanib. However, a prospective cohort study found that the more frequent bowel movements, the higher the risk of diverticulitis (Jovani et al., 2022). Therefore, the diarrhea associated with nintedanib use may be related to the positive signal of diverticulitis observed in this study.

Surprisingly, our SOC-level analysis revealed that “skin and subcutaneous tissue disorders” accounted for a higher proportion and represented a significant SOC for pirfenidone. The most common treatment-emergent adverse events with pirfenidone were photosensitivity reactions (*n* = 634, ROR 28.85 [26.62–31.27]), which were generally mild to moderate, as reported in both the CAPACITY and ASCEND studies (Kim and Keating, 2015). A 64-year-old male patient treated with pirfenidone developed a severe phototoxic reaction that disappeared after subsequent glucocorticoid therapy (Papakonstantinou et al., 2016). A case series study found that of 54 patients treated with pirfenidone, 13 (22.2%) developed skin symptoms (Droitcourt et al., 2018). A nationwide post-marketing surveillance study on IPF patients in Korea reported photosensitivity reactions (13.7%) to be the most common reactions (Chung et al., 2020). Sunburn (*n* = 326, EBGM05 = 28.84) is an ADE not mentioned in the drug label, which may be due to the different terminologies used by reporters to describe photosensitivity reactions. Photosensitivity increases the skin’s sensitivity to sunlight or ultraviolet (UV) radiation. This means that patients taking pirfenidone are more vulnerable to UV-induced skin damage when exposed to sunlight, leading to sunburn. Furthermore, pirfenidone treatment downregulates COL1A1, a gene involved in the production of collagen for wound healing (Ishii et al., 2024). As a result, long-term use of pirfenidone, particularly in patients requiring skin repair, may impair the skin’s healing process and lead to slower recovery from sunburn. During treatment, patients should avoid prolonged sun exposure, especially during periods of intense sunlight, and are advised to adopt appropriate protective measures, such as using sunscreen and wearing long-sleeved clothing.

For nintedanib, “investigations” and “hepatobiliary disorders” accounted for higher proportions of ADEs and represented significant SOCs. Weight decreased (*n* = 2,153, ROR 5.19 [4.97–5.41]), an adverse effect during nintedanib treatment and a common complication that can be used as a prognostic indicator, was the most common PT at the SOC level of examination (Tomioka et al., 2023). A retrospective study also predicted a higher weight loss with nintedanib than with pirfenidone (Perelas et al., 2019). Therefore, patients must control their weight and prevent weight loss. In our study, hepatobiliary disorders (n = 251, ROR 4.14 [3.65–4.69]) accounted for a higher proportion of ADE reports in nintedanib compared to pirfenidone. Fortunately, the clinical consequences are minimal and can be reversed by dose reduction or discontinuation (Richeldi et al., 2014). However, the results of the phase III LUME-Colon 1 trial found that the most common grade ≥3 adverse events were those related to the liver (nintedanib 16%; placebo 8%) (Van Cutsem et al., 2018). Notably, Raschi et al. found that concomitant drugs with hepatotoxicity were documented in almost half of the cases (Raschi et al., 2022). Hence, it is necessary to pay attention to individual differences in liver dysfunction when using nintedanib.

### 4.3 Signal detection at PT

The signal strength of ADEs at the PT level of both drugs was systematically examined and ranked according to their frequencies and EBGM05 values. By comparing with the drug label, we identified several unexpected and positive signals not listed in the drug labels. For pirfenidone, unexpected signals with a large number of cases included pneumothorax [*n* = 130, EBMG05 (Empirical Bayes Geometric Mean 5th percentile) = 4.99), arteriosclerosis coronary artery (*n* = 44, EBMG05 = 4.73) and gastric pH decreased (*n* = 7, EBMG05 = 2.55). Our findings suggest that pneumothorax is a common and unexpected signal. Pirfenidone primarily mitigates fibrosis through its anti-inflammatory effects, but there is currently no conclusive evidence to suggest that it directly causes the occurrence of pneumothorax. The Japanese Real Clinical World study found one case of pneumothorax, a serious adverse event, resulting from the combination of pirfenidone and nintedanib (Hisata et al., 2021). Therefore, we need to pay close attention to this side effect, particularly if unexplained chest pain or worsening shortness of breath occurs. Previous studies have found that coronary artery disease significantly negatively impacts the survival rate of patients with IPF(Kreuter et al., 2016). Animal studies have shown that pirfenidone offers protective effects in a rat model of cardiac fibrosis, potentially mediated by a feedback loop involving the angiotensin II type 1 receptor/p38 mitogen-activated protein kinase/renin-angiotensin system axis, which is regulated through liver X receptor-alpha (Li et al., 2017). Furthermore, a study from three Phase III trials of pirfenidone in IPF patients found that pirfenidone didn’t increase the risk of cardiovascular events (Glassberg et al., 2019). However, these results are limited to a carefully selected population in clinical trials. Therefore, our real-world data reveal that arteriosclerotic coronary artery disease (n = 44, EBMG05 = 4.73) may be an unexpected signal potentially associated with pirfenidone use, but further clinical trials are needed to explore and validate the potential relationship. Gastrointestinal ADEs are commonly observed in patients with IPF treated with pirfenidone (Molina-Molina et al., 2023). Our data suggest that decreased gastric pH (n = 7, EBMG05 = 2.55) may be one of the factors contributing to these gastrointestinal ADEs. Increased gastric acid secretion leads to decreased gastric pH, which can exacerbate discomfort and create a vicious cycle, further triggering gastrointestinal symptoms. This is particularly relevant for patients with pre-existing chronic gastric conditions, where timely adjustments to the treatment plan or measures to alleviate gastrointestinal symptoms may be necessary to improve the patient’s medication experience. In addition, we found some unexpected signals with high EBGM05 values, such as lung transplant (*n* = 80, EBGM05 = 11.1), increased sputum (*n* = 37, EBGM05 = 8.17), and increased oxygen consumption (*n* = 40, EBGM = 7.45). We also identified a number of PTs with positive signal values but relatively small numbers, such as defaecation disorder (n = 8, ROR 6.59 [3.28–13.24]), catarrh (n = 7, ROR 4.61 [2.19–9.7]), and hemiplegic migraine (n = 5, ROR 9.77 [4.03–23.69]); These signals are not listed on the drug label. However, considering our current exploratory study based on pharmacovigilance analysis, their potential association with pirfenidone use may require further validation in future clinical trials.

Regarding nintedanib, unexpected signals with a large number of cases have been reported, which include constipation (*n* = 1,385, EBMG05 = 4.02), flatulence (*n* = 718, EBMG05 = 8.35), and haematochezia (*n* = 389, EBMG05 = 4.05). Our data suggest that nintedanib might be associated with constipation, potentially due to its correlation with the increased use of anti-diarrhea medications in the management of diarrhea. However, our sensitivity analyses, which excluded the potential interference of other medications, still indicate a positive signal for constipation. Although the association between constipation, flatulence, and nintedanib use requires further evaluation, patients undergoing nintedanib treatment should be vigilant about the potential concomitant risks of constipation and flatulence. Some unexpected signals with high EBGM05 values have also been reported, such as increased oxygen consumption (*n* = 109, EBMG05 = 21.35), lung transplant (n = 172, EBMG05 = 24.03), and abnormal oxygen saturation (*n* = 56, EBMG05 = 9.73). The high-strength signal associated with nintedanib is primarily caused by hypoxia. Hence, physicians should closely monitor the oxygen saturation and lung function of patients on nintedanib. Importantly, nintedanib inhibits the VEGFR and epidermal growth factor receptor (EGFR), potentially leading to vascular dysfunction and increased risk of bleeding (Roodhart et al., 2008; Touyz et al., 2018). Our study identified several types of bleeding side effects not listed on the drug label, such as rectal haemorrhage (n = 261, EBMG05 = 3.77), haemoptysis (n = 243, EBMG05 = 5.35), haemorrhage (n = 104, EBMG05 = 3.76), and diarrhoea haemorrhagic (n = 93, EBMG05 = 5.66). A patient suffering from colitis due to nintedanib experienced haematochezia and diarrhoea, which were relieved after using budesonide (Amini et al., 2020). Studies to evaluate the long-term safety and tolerability of nintedanib in patients with IPF have reported a bleeding rate of 8.4 cases per 100 patient-years of exposure for patients continuing nintedanib and 6.7 cases per 100 patient-years of exposure for patients initiating nintedanib (Crestani et al., 2019). A 74-year-old patient developed acute aortic syndrome with intramural haematoma (IMH) during treatment with the tyrosine kinase inhibitor nintedanib. Four months after discontinuation of the drug, the IMH had significantly regressed (Willems et al., 2021). Real-world data from EMPIRE suggest that nintedanib is less frequently taken by patients receiving anticoagulants, but bleeding events are not associated with the combination of antifibrotic drugs with anticoagulant or antiplatelet therapy (Kolonics-Farkas et al., 2020). In addition, an observational study found that antifibrotic drugs do not increase the risk of postoperative bleeding after lung transplantation in patients with IPF(Moncomble et al., 2024). Our findings indicate that the incidence of drug-related adverse bleeding events associated with nintedanib still requires vigilance and should be further verified in future clinical trials. Hypogeusia (n = 18, EBMG05 = 4.53) can lead to a loss of interest in food and inadequate nutrient intake. In another pharmacovigilance analysis, it was also found that nintedanib was most strongly associated with taste and smell disorders [PRR = 4.73, ROR = 4.82, information component (IC) = 2.23], but the mechanism between them is not yet clear (Fu et al., 2025). In our study, although some newly identified signals have not been listed in the drug label, they may represent specific manifestations of signals already included in the label. It is important to note that our findings primarily serve as recommendations for future observational studies, aimed at further evaluating these potential risks. While the current results do not establish a causal relationship between antifibrotic drugs and the associated adverse events, they provide clinicians with detailed information regarding the potential adverse reactions of these two drugs. Future clinical trials will help validate the relationship between these signals, and if necessary, may prompt updates to the drug labeling.

### 4.4 TTO analysis

The temporal relationship between drug administration and the onset time of ADEs is crucial for assessing drug safety (Zou F. et al., 2024). Previous clinical studies have provided a wealth of information on ADEs. However, the exact timing of these events remains largely unknown. Our results showed that most pirfenidone-related adverse events occurred in the first month (20.26%) and over 1 year (28.80%). In contrast, most nintedanib-related adverse events occurred in the first month (42.11%); only 16.28% occurred after 1 year. The main indication for antifibrotic drugs is IPF, which requires the long-term use of antifibrotic drugs. However, this can lead to undesirable side effects, which can occur at any time during the treatment. Fortunately, the Weibull distribution test showed a decrease in the likelihood of ADEs over time for both drugs, a result that is encouraging for drugs that require long-term use. We found that the median TTO of nintedanib-related ADEs (45 days) was earlier than pirfenidone (146 days) (Figures 5E,F). At the SOC level, in terms of gastrointestinal disorders, the median TTO of nintedanib-associated diarrhea was 35 days, with many PTs occurring within the first month of treatment. In contrast, pirfenidone-associated gastrointestinal ADEs (e.g., nausea and diarrhea) typically have a later onset, with a median onset time of more than 1 month. Compared with pirfenidone, nintedanib showed earlier onset of ADEs associated with hepatobiliary disorders, for example drug-related liver injury, with a median onset time of 12 days. Importantly, we provided detailed TTO data for all SOCs and PTs for each drug SOC of interest (Supplementary Tables S4–S6). A clinical trial found a significantly higher incidence of discontinuations in the nintedanib group than in the pirfenidone group within 1 year of therapy (76% versus 37%) because of adverse events (Takehara et al., 2022). However, doctors usually ask patients to start taking pirfenidone at a low dose and then gradually increase the dose to minimize the risk of ADEs. This titration may account for the longer time it takes for ADEs to occur with pirfenidone, as gradually increasing the dose allows the body to adjust, thereby delaying the onset of ADEs.

Any guidance that helps the patient continue treatment is likely to improve the outcome (Bendstrup et al., 2019; Rahaghi et al., 2020). Previous investigators have reported the median onset time for some specific ADEs with pirfenidone dosing. It has also been reported that dermatological ADEs occur within the first 2–3 months of treatment (Lancaster et al., 2017). Our study also reached similar conclusions. Gastrointestinal disorders are one of the side effects listed in pirfenidone labeling. These side effects occurred mainly within the first 2–3 months of treatment, except for dry mouth and dysphagia (Supplementary Tables S4–S6). The remaining four SOCs (Figures 6B–E) also showed greater variability in the PT-onset time. However, the median onset time of the specific ADEs of nintedanib is not sufficiently investigated. Unlike pirfenidone, all gastrointestinal side effects observed associated with nintedanib occurred within 1–2 months of treatment. Notably, PTs related to hepatobiliary diseases other than cholelithiasis and jaundice occurred mainly in the first month (Supplementary Tables S6). Therefore, early observation of gastrointestinal disorders caused by nintedanib, monitoring the patient’s liver function, and timely conducting abdominal ultrasound scans may prove useful in the early detection of common side effects associated with this drug. For selecting different antifibrotic drugs, the requirements for early detection and tracking of side effects in different systems should be different.

### 4.5 Validation of results in JADER database

Regarding basic epidemiologic characteristics, such as sex distribution, age and indications, the results of the JADER database are generally consistent with FAERS, which strengthens the credibility of our results. Data from FAERS and JADER may vary by race, social background, and medical condition (Hosomi et al., 2015). At the SOC level, “respiratory, thoracic, and mediastinal disorders”, “metabolism and nutrition disorders” are significant SOCs for pirfenidone in both databases. “Hepatobiliary disorders” is the unique significant SOC for pirfenidone in JADER database. For nintedanib, “respiratory, thoracic, and mediastinal disorders”, “metabolism and nutrition disorders” and “hepatobiliary disorders” are significant SOCs in both databases. Similar to the results in FAERS, decreased appetite (n = 33, ROR 12.57) and photosensitivity reaction (n = 19, ROR 114.56) were among the top 50 highest percentage PT entries in the FAERS for pirfenidone. For nintedanib, diarrhea (n = 101, ROR 6.97), decreased appetite (n = 84, ROR 6.83), hepatic function abnormality (n = 77, ROR 4.25), and drug-induced liver injury (n = 37, ROR 4.75) were among the top 50 highest percentage PT entries. For unexpected PTs, pneumothorax (n = 19, ROR 48.63) with pirfenidone, and pneumothorax (n = 82, ROR 43.35), pneumatosis intestinal (n = 13, ROR 8.47), and taste disorder (n = 5, ROR 7.23) with nintedanib were also observed in the JADER database. These results are similar in the two databases, which further enhances the reliability of our results. However, due to regional differences between the two databases and limitations in sample size, we must also acknowledge the existence of unique differences (Imai et al., 2022). For example, hepatic function abnormality (n = 21, ROR 4.54) is a common ADE of pirfenidone in the JADER data and was not observed in FAERS. This may be because these adverse reactions are common with adverse reaction reports for other drugs in the FAERS database, which in turn affects the signal value. Disproportionate requires a higher (or lower) frequency of ADE reports for certain drugs. The absence of a signal does not mean that there are no relative ADEs, but only that these side effects are not disproportionately common. To better supplement our findings, we further validated the ADEs in JADER that were strongly associated with the two antifibrotic drugs. Photosensitivity reaction and pneumothorax for pirfenidone, and lung transplant and lung surgery for nintedanib could also be observed in FAERS. Due to the limited sample size, the TTO analysis at the PT level showed that there was no significant difference among the 9 PTs taking pirfenidone. For nintedanib, we observed that the TTO for hepatic function abnormal and drug-induced liver injury in the two databases were very close. However, in JADER, the median TTO of pneumothorax was the longest [205.5 days (IQR 85.5–396.75 days)], but in FAERS, the median TTO was 42 days (IQR 16–119.5 days).

### 4.6 Limitations

Although this study provides a scientific analysis of real-world data on the safety of the two antifibrotic drugs from multiple perspectives, it also has the following limitations.

#### 4.6.1 Impact of potential diseases and concomitant medications

Due to the reliance on voluntary reporting in the FAERS database, incomplete reporting may occur, particularly in the absence of detailed clinical information about patients (such as underlying diseases, comorbidities, and concomitant medications) (Liu F. et al., 2024). Our analysis suggests that nintedanib may have potential hepatotoxicity, and the risk of drug-induced liver injury may be higher in patients with a history of liver disease. However, the FAERS database lacks detailed patient history, making it difficult to fully control for the confounding effect of underlying diseases on the outcomes. Moreover, during the treatment of IPF, patients often require additional medications to manage associated symptoms or comorbid conditions, and certain concomitant drugs may either mask or amplify the potential side effects of antifibrotic therapies. Despite conducting sensitivity analyses, these confounding factors should be carefully considered, as they may lead to bias in pharmacovigilance analysis results.

#### 4.6.2 Impact of healthcare system differences

Differences in healthcare systems, including accessibility to healthcare services, variations in clinical practices, differences in drug monitoring systems, demographic disparities, and variations in adverse event reporting mechanisms, may significantly affect the consistency of drug safety reports and evaluations, thereby influencing pharmacovigilance analysis results (Menang et al., 2023). Therefore, when interpreting and comparing results from different databases, careful consideration of these systemic differences and their potential impact is essential.

#### 4.6.3 Lower reporting rates of certain adverse events in the JADER database

In the FAERS database, patients can report adverse events through various channels (e.g., drug manufacturer websites or FDA platforms). In contrast, in Japan, patients typically report through healthcare providers such as physicians or pharmacists. This reporting structure has led to a significant difference in case numbers between the two databases, with the JADER database reporting fewer than one-tenth of the cases found in FAERS. In pharmacovigilance analyses, lower reporting rates of certain adverse events may impair the comprehensive understanding of a drug’s side effects, thereby affecting the accuracy of drug safety assessments (Zou et al., 2024b).

#### 4.6.4 Inherent biases in spontaneous reporting systems

In spontaneous reporting databases, reports are typically voluntary, meaning not all adverse events are reported (Zhang et al., 2024). Selective reporting may lead to the underestimation of mild, non-severe, or short-term side effects. Furthermore, incomplete reporting (such as missing critical information such as age, sex, drug dosage, and treatment duration) may hinder clinicians from making accurate drug risk assessments (Hu et al., 2025). Reporting bias may affect data quality, with some adverse events being over-reported due to market attention or media coverage, leading to an overestimation of drug risks. Conversely, some rare but severe side effects may not be adequately recorded due to their low incidence (Liu et al., 2025). For example, in this study, consumers were the primary reporters in the FAERS database, and given their lack of professional medical knowledge, this may introduce reporting bias. As a result, the incompleteness of these reports may lead to analysis results that do not accurately reflect the true safety of the drug.

#### 4.6.5 Unexpected positive signals may reflect disease progression rather than drug effects

Although our study identified several positive signals not listed in the drug label, these signals could also be a result of disease progression rather than a direct manifestation of the drug’s effect. Particularly in chronic diseases like IPF, patients’ conditions may naturally deteriorate over time. Disease progression may confound the interpretation of adverse drug reactions or therapeutic efficacy, and further research is needed to distinguish between the effects of the drug and the progression of the disease itself (Xing et al., 2025).

#### 4.6.6 Causality cannot be determined

Signal detection in pharmacovigilance studies only reflects statistical associations, providing estimates of signal strength rather than direct causality. Therefore, prospective clinical studies are required to confirm the causal relationships of these associations (Gong et al., 2025).

#### 4.6.7 Generalizability of conclusions to other populations

This study primarily relied on data from the United States (FAERS) and Japan (JADER), which may limit the generalizability of the findings to other populations, especially given the differences in population characteristics, medical practices, and prescribing patterns (Liu Q. et al., 2024).

## 5 Conclusion

**FIGURE 9:**
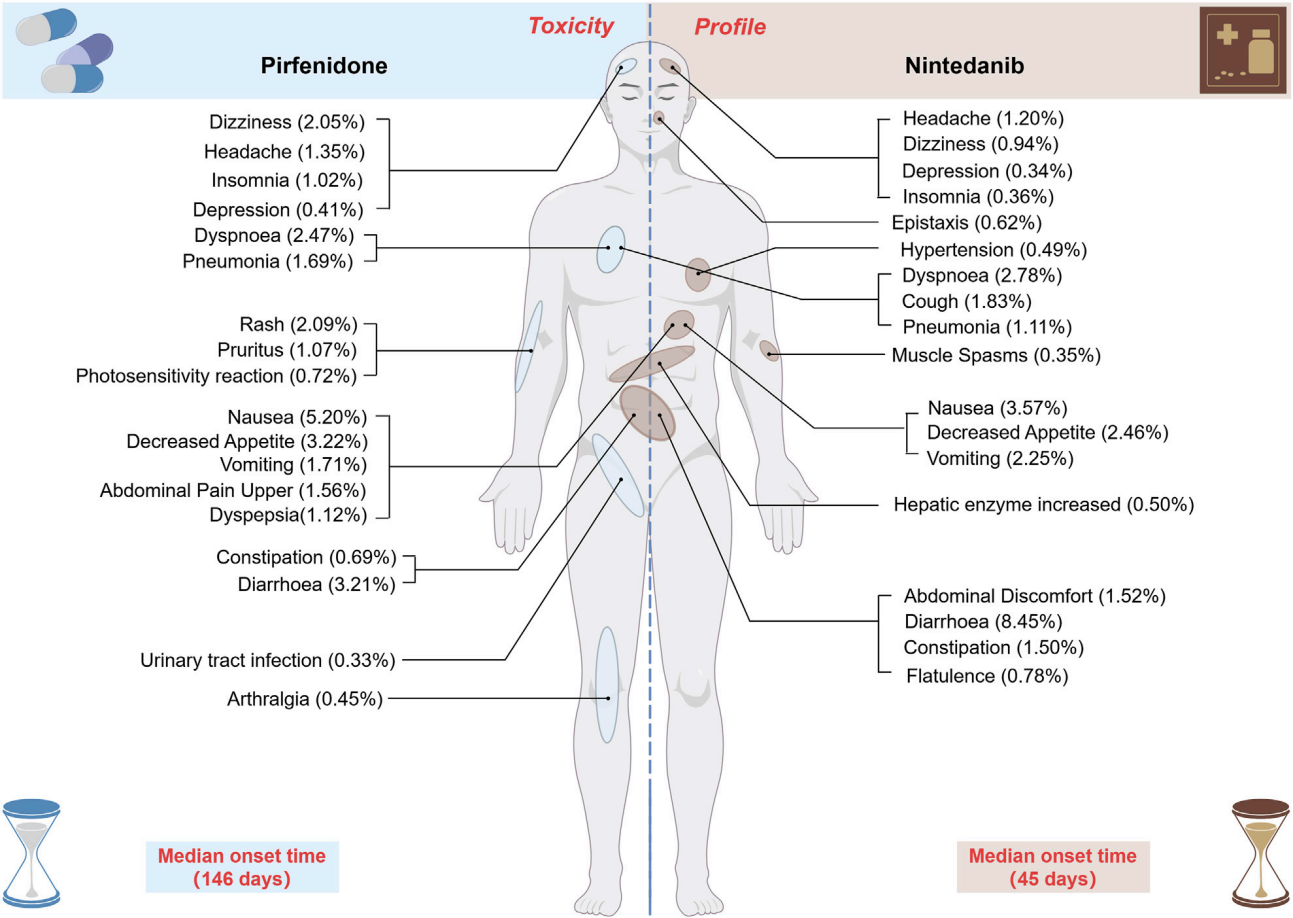
Toxicity profile of the two antifibrotic drugs (pirfenidone and nintedanib) in FAERS. The figure shows the reporting frequency of the given PT in the two drugs, along with the median time of adverse event occurrence.

We utilized the latest FAERS and JADER data to conduct a multi-dimensional and multi-level analysis, comparing the safety of these two drugs using four methods of disproportionality analysis. Some positive signals were consistent with the drug labels, including nausea, decreased appetite, and weight decreased identified in pirfenidone, as well as diarrhea, decreased appetite, upper abdominal pain, and epistaxis identified in nintedanib. Additionally, we identified unexpected signals not listed on the drug label, such as decreased gastric pH and pneumothorax for pirfenidone, and constipation and flatulence for nintedanib. Moreover, the median onset times for ADEs were 146 days for pirfenidone and 45 days for nintedanib, showing early failure type, which indicates that the risk of adverse events decreases over time. Figure 9 summarizes the key findings from our analysis of the FAERS database. In the JADER database, we identified 14 positive PTs for pirfenidone and 32 for nintedanib, most of which were consistent with the results from FAERS. Although our findings provide interesting reference information on the safety of clinical antifibrotic drugs, it is essential to interpret these results cautiously due to the limitations of the data. Furthermore, to establish the clinical relevance of the signals identified in this study, we recommend conducting well-designed observational studies to validate these findings. Prospective clinical trials should follow once these signals are confirmed.

## Data Availability

The original contributions presented in the study are included in the article/Supplementary Material, further inquiries can be directed to the corresponding author.

## Supplementary material

SUPPLEMENTARY TABLE S1 In pirfenidone, 87 positive signals satisfy thresholds of the four methods of disproportionality analysis. PT entries are displayed in the descending order of case numbers. Signals not listed on the drug label are marked with asterisks. SOC, system organ class; PT, preferred term.

SUPPLEMENTARY TABLE S2 In nintedanib, 176 positive signals satisfy thresholds of four methods of disproportionality analysis simultaneously. PT entries are displayed in the descending order of case numbers. Signals not listed on the drug label are marked with asterisks. SOC, system organ class; PT, preferred term.

SUPPLEMENTARY TABLE S3 Sensitivity analysis results. All positive signals for pirfenidone and nintedanib were derived from the FAERS data (the effect of concomitant medication on the results was eliminated at the PT level). SOC, system organ class; PT, preferred term.

SUPPLEMENTARY TABLE S4 Detailed TTO analysis at the SOC levels. n, reported cases; Min, minimum; Max: maximum; IQR, interquartile range; q1, 1/4 quantile; q3, 3/4 quantile; SD, standard deviation; SE, standard error.

SUPPLEMENTARY TABLE S5 Comprehensive TTO analysis at the PT level for pirfenidone. No less than 20 cases of PT are selected for analysis under each positive SOC. n, reported cases; Min, minimum; Max: maximum; IQR, interquartile range; q1, 1/4 quantile; q3, 3/4 quantile; SD, standard deviation; SE, standard error. SOC, system organ class; PT, preferred term.

SUPPLEMENTARY TABLE S6 Comprehensive TTO analysis at the PT level for nintedanib. No less than 20 cases of PT are selected for analysis under each positive SOC. n, reported cases; Min, minimum; Max: maximum; IQR, interquartile range; q1, 1/4 quantile; q3, 3/4 quantile; SD, standard deviation; SE, standard error. SOC, system organ class; PT, preferred term.

SUPPLEMENTARY TABLE S7 Validation results in JADER database. JADER, the Japanese Adverse Drug Event Report database.
