# Supplementary material for: Post-marketing safety concerns with pirfenidone and nintedanib: an analysis of individual case safety reports from the FDA adverse event reporting system database and the Japanese adverse drug event report databases
Source: Front Pharmacol. 2025 Apr 28;16:1530697. doi: 10.3389/fphar.2025.1530697 (PMC12067420; doi:10.3389/fphar.2025.1530697)
Supplement: Supplementary file 1 [file DataSheet1.zip › Supplementary table 7.DOCX]

Number of reported ADEs per year for two antifibrotic drugs in the JADER database

| Year | 2008 | 2009 | 2010 | 2011 | 2012 | 2013 | 2014 | 2015 | 2016 | 2017 | 2018 | 2019 | 2020 | 2021 | 2022 | 2023 | 2024(Q1) |
| --- | --- | --- | --- | --- | --- | --- | --- | --- | --- | --- | --- | --- | --- | --- | --- | --- | --- |
| pirfenidone | 2 | 52 | 52 | 39 | 19 | 15 | 16 | 18 | 10 | 13 | 12 | 9 | 3 | 5 | 7 | 4 | 0 |
| ninetanib | NA | NA | NA | NA | NA | NA | NA | 42 | 155 | 168 | 165 | 121 | 121 | 90 | 223 | 208 | 34 |

Description of baseline information for reported ADEs for pirfenidone.

| **Characteristics** | **Case number** | **Case proportion, %** |
| --- | --- | --- |
| **Gender, n (%)** | 276 |  |
| Female | 54 | 19.6% |
| Male | 215 | 77.9% |
| Unknown | 7 | 2.5% |
| **Age (years)**  <20 | 1 | 0.4% |
| 20-70 | 225 | 81.5% |
| >70 | 31 | 11.2% |
| Unknown | 19 | 6.9% |
| **Weight (kg)**  <50 | 21 | 7.6% |
| 50-100 | 67 | 24.3% |
| >100 | 0 | 0% |
| Unknown | 188 | 68.1% |
| **Outcome** |  |  |
| Recovery (recovery but with sequelae) | 4 | 1.0% |
| Rehabilitation | 149 | 36.7% |
| Minor rehabilitation | 97 | 23.9% |
| Death | 49 | 12.1% |
| Non-rehabilitated | 74 | 18.2% |
| Missing | 33 | 8.1% |
| **Indication (top three)** |  |  |
| idiopathic pulmonary fibrosis | 199 | 72.1% |
| interstitial lung disease | 34 | 12.3% |
| Others | 43 | 15.6% |

Description of baseline information for reported ADEs for nintedanib.

| **Characteristics** | **Case number** | **Case proportion, %** |
| --- | --- | --- |
| **Gender, n (%)** | 1327 |  |
| Female | 332 | 25.0% |
| Male | 970 | 73.1% |
| Unknown | 25 | 1.9% |
| **Age (years)**  <20 | 1 | 0.1% |
| 20-70 | 1047 | 78.9% |
| >70 | 219 | 16.5% |
| Unknown | 60 | 4.5% |
| **Weight (kg)**  <50 | 223 | 16.8% |
| 50-100 | 544 | 41.0% |
| >100 | 0 | 0% |
| Unknown | 560 | 42.2% |
| **Outcome** |  |  |
| Recovery (recovery but with sequelae) | 23 | 1.2% |
| Rehabilitation | 787 | 39.8% |
| Minor rehabilitation | 167 | 8.5% |
| Death | 297 | 15.0% |
| Non-rehabilitated | 307 | 15.5% |
| Missing | 394 | 19..9% |
| **Indication (top three)** |  |  |
| idiopathic pulmonary fibrosis | 916 | 69.0% |
| interstitial lung disease | 292 | 22.0% |
| Others | 119 | 9.0% |

Signal detection at the SOC level for both drugs. Numbers 1 and 2 marked in the lower right corner refer to calculations related to pirfenidone and nintedanib, respectively. ADE, adverse drug event.

| **SOC name** | Case number1 | Case number2 | ROR(95%CI)1 | ROR(95%CI)2 | PRR(χ2)1 | PRR(χ2)2 | EBGM(EBGM05)1 | EBGM(EBGM05)2 | IC(IC025)1 | IC(IC025)2 |
| --- | --- | --- | --- | --- | --- | --- | --- | --- | --- | --- |
| Respiratory, thoracic and mediastinal disorders | 96 | 499 | 3.98(3.16-5) | 4.26(3.85-4.72) | 3.27(163.06) | 3.44(924.35) | 3.27(163.06) | 3.42(3.09) | 3.27(2.6) | 1.77(0.11) |
| Gastrointestinal disorders | 36 | 291 | 1.12(0.8-1.58) | 2.03(1.79-2.3) | 1.11(0.44) | 1.88(129.77) | 1.11(0.44) | 1.88(1.66) | 1.11(0.79) | 0.91(-0.76) |
| General disorders and administration site conditions | 32 | 191 | 1.17(0.82-1.69) | 1.37(1.18-1.59) | 1.16(0.76) | 1.33(17) | 1.16(0.76) | 1.33(1.15) | 1.16(0.81) | 0.41(-1.25) |
| Hepatobiliary disorders | 33 | 179 | 2.36(1.65-3.37) | 3.02(2.59-3.52) | 2.25(23.71) | 2.84(218.46) | 2.25(23.71) | 2.82(2.42) | 2.25(1.57) | 1.5(-0.17) |
| Investigations | 42 | 142 | 1.13(0.82-1.56) | 0.88(0.74-1.04) | 1.12(0.57) | 0.89(2.28) | 1.12(0.57) | 0.89(0.75) | 1.12(0.81) | -0.17(-1.84) |
| Metabolism and nutrition disorders | 38 | 124 | 2.29(1.64-3.19) | 1.52(1.26-1.82) | 2.17(24.93) | 1.49(20.45) | 2.17(24.93) | 1.48(1.24) | 2.17(1.55) | 0.57(-1.1) |
| Infections and infestations | 30 | 117 | 0.88(0.61-1.28) | 0.7(0.58-0.84) | 0.89(0.42) | 0.71(14.57) | 0.89(0.42) | 0.71(0.59) | 0.89(0.62) | -0.48(-2.15) |
| Neoplasms benign, malignant and unspecified (incl cysts and polyps) | 9 | 77 | 0.52(0.27-1) | 0.79(0.63-0.99) | 0.53(3.93) | 0.8(4.05) | 0.53(3.93) | 0.8(0.64) | 0.53(0.27) | -0.32(-1.99) |
| Nervous system disorders | 14 | 70 | 0.34(0.2-0.57) | 0.34(0.27-0.43) | 0.36(17.6) | 0.36(85.69) | 0.36(17.6) | 0.37(0.29) | 0.36(0.21) | -1.45(-3.12) |
| Cardiac disorders | 15 | 56 | 0.89(0.53-1.5) | 0.64(0.49-0.84) | 0.9(0.19) | 0.65(10.87) | 0.9(0.19) | 0.65(0.5) | 0.9(0.54) | -0.62(-2.28) |
| Renal and urinary disorders | 3 | 44 | 0.18(0.06-0.56) | 0.54(0.4-0.72) | 0.19(11.03) | 0.55(17.14) | 0.19(11.03) | 0.55(0.41) | 0.19(0.06) | -0.87(-2.54) |
| Blood and lymphatic system disorders | 17 | 40 | 0.66(0.4-1.07) | 0.33(0.24-0.45) | 0.67(2.92) | 0.34(54.24) | 0.67(2.92) | 0.34(0.25) | 0.67(0.41) | -1.55(-3.22) |
| Musculoskeletal and connective tissue disorders | 3 | 33 | 0.27(0.09-0.84) | 0.6(0.43-0.85) | 0.28(5.86) | 0.61(8.42) | 0.28(5.86) | 0.61(0.43) | 0.28(0.09) | -0.71(-2.38) |
| Vascular disorders | 6 | 21 | 0.55(0.25-1.24) | 0.38(0.25-0.58) | 0.56(2.13) | 0.39(21.02) | 0.56(2.13) | 0.39(0.25) | 0.56(0.25) | -1.37(-3.04) |
| Surgical and medical procedures | #N/A | 21 | #N/A | 1.84(1.2-2.83) | #N/A | 1.83(7.95) | #N/A | 1.83(1.19) | #N/A | 0.87(-0.8) |
| Skin and subcutaneous tissue disorders | 18 | 17 | 0.74(0.46-1.19) | 0.16(0.1-0.25) | 0.75(1.56) | 0.16(75.95) | 0.75(1.56) | 0.16(0.1) | 0.75(0.47) | -2.6(-4.27) |
| Injury, poisoning and procedural complications | #N/A | 17 | #N/A | 0.24(0.15-0.39) | #N/A | 0.25(39.65) | #N/A | 0.25(0.16) | #N/A | -2(-3.67) |
| Psychiatric disorders | 3 | 9 | 0.36(0.12-1.13) | 0.25(0.13-0.48) | 0.37(3.31) | 0.25(20.07) | 0.37(3.31) | 0.25(0.13) | 0.37(0.12) | -1.98(-3.64) |
| Eye disorders | 3 | 7 | 0.47(0.15-1.48) | 0.22(0.1-0.45) | 0.48(1.73) | 0.22(19.78) | 0.48(1.73) | 0.22(0.1) | 0.48(0.15) | -2.19(-3.85) |
| Immune system disorders | #N/A | 5 | #N/A | 0.09(0.04-0.21) | #N/A | 0.09(47.9) | #N/A | 0.09(0.04) | #N/A | -3.49(-5.15) |
| Reproductive system and breast disorders | #N/A | 5 | #N/A | 0.65(0.27-1.56) | #N/A | 0.65(0.96) | #N/A | 0.65(0.27) | #N/A | -0.63(-2.29) |
| Endocrine disorders | 4 | 4 | 0.56(0.21-1.51) | 0.09(0.03-0.24) | 0.57(1.33) | 0.09(37.33) | 0.57(1.33) | 0.09(0.03) | 0.57(0.21) | -3.46(-5.13) |

For pirfenidone, 14 positive signals satisfy the thresholds of four methods of disproportionality analysis simultaneously. PT entries are displayed in the descending order of case numbers. Signals not listed on the drug label are marked with asterisks.

| soc_name | pt_name | a | ROR(95%CI) | PRR(χ2) | EBGM(EBGM05) | IC(IC025) | Adjusted-P |
| --- | --- | --- | --- | --- | --- | --- | --- |
| Metabolism and nutrition disorders | Decreased appetite | 33 | 12.57 ( 8.8 - 17.95 ) | 11.63 ( 321.68 ) | 11.59 ( 8.11 ) | 3.53 ( 1.86 ) | 1.89E-68 |
| Hepatobiliary disorders | Hepatic function abnormal | 21 | 4.54 ( 2.92 - 7.04 ) | 4.35 ( 54.82 ) | 4.35 ( 2.8 ) | 2.12 ( 0.45 ) | 1.08E-11 |
| Respiratory, thoracic and mediastinal disorders | Pneumothorax* | 19 | 48.63 ( 30.59 - 77.32 ) | 46.4 ( 833.27 ) | 45.78 ( 28.79 ) | 5.52 ( 3.84 ) | 1.96E-172 |
| Skin and subcutaneous tissue disorders | Photosensitivity reaction | 11 | 114.56 ( 62.32 - 210.6 ) | 111.49 ( 1165.53 ) | 107.89 ( 58.69 ) | 6.75 ( 5.07 ) | 1.46E-231 |
| Blood and lymphatic system disorders | Agranulocytosis | 8 | 8.32 ( 4.13 - 16.76 ) | 8.17 ( 50.36 ) | 8.16 ( 4.05 ) | 3.03 ( 1.35 ) | 6.55E-10 |
| Respiratory, thoracic and mediastinal disorders | Pneumonitis* | 6 | 5.69 ( 2.54 - 12.75 ) | 5.62 ( 22.82 ) | 5.61 ( 2.51 ) | 2.49 ( 0.82 ) | 0.00026474 |
| Respiratory, thoracic and mediastinal disorders | Lung disorder* | 5 | 7.22 ( 2.99 - 17.46 ) | 7.14 ( 26.4 ) | 7.13 ( 2.95 ) | 2.83 ( 1.16 ) | 8.41E-05 |
| Endocrine disorders | Inappropriate antidiuretic hormone secretion* | 4 | 7.04 ( 2.63 - 18.88 ) | 6.98 ( 20.5 ) | 6.97 ( 2.6 ) | 2.8 ( 1.13 ) | 0.001655144 |
| Investigations | Gamma-glutamyltransferase increased | 3 | 9.01 ( 2.89 - 28.09 ) | 8.95 ( 21.14 ) | 8.93 ( 2.86 ) | 3.16 ( 1.48 ) | 0.002814267 |
| Infections and infestations | Bronchopulmonary aspergillosis* | 3 | 9.02 ( 2.89 - 28.14 ) | 8.97 ( 21.19 ) | 8.94 ( 2.87 ) | 3.16 ( 1.49 ) | 0.002765497 |
| Respiratory, thoracic and mediastinal disorders | Pulmonary haemorrhage* | 3 | 18.14 ( 5.81 - 56.65 ) | 18.01 ( 47.96 ) | 17.92 ( 5.74 ) | 4.16 ( 2.49 ) | 1.77E-07 |
| Neoplasms benign, malignant and unspecified (incl cysts and polyps) | Lung neoplasm malignant* | 3 | 8.31 ( 2.67 - 25.92 ) | 8.26 ( 19.11 ) | 8.24 ( 2.64 ) | 3.04 ( 1.37 ) | 0.005957621 |
| Respiratory, thoracic and mediastinal disorders | Pneumomediastinum* | 3 | 41.04 ( 13.09 - 128.68 ) | 40.75 ( 114.93 ) | 40.27 ( 12.84 ) | 5.33 ( 3.65 ) | 9.32E-18 |
| Hepatobiliary disorders | Hepatitis fulminant* | 3 | 11.61 ( 3.72 - 36.23 ) | 11.53 ( 28.78 ) | 11.5 ( 3.69 ) | 3.52 ( 1.85 ) | 0.000173585 |

For nintedanib, 32 positive signals satisfy the thresholds of four methods of disproportionality analysis simultaneously. PT entries are displayed in the descending order of case numbers. Signals not listed on the drug label are marked with asterisks.

| soc_name | pt_name | a | ROR(95%Cl) | PRR(χ2) | EBGM(EBGM05) | IC(IC025) | Adjusted-P |
| --- | --- | --- | --- | --- | --- | --- | --- |
| General disorders and administration site conditions | Death* | 108 | 6.71 ( 5.52 - 8.16 ) | 6.4 ( 489.83 ) | 6.33 ( 5.21 ) | 2.66 ( 0.99 ) | 8.69E-106 |
| Gastrointestinal disorders | Diarrhoea | 101 | 6.97 ( 5.7 - 8.53 ) | 6.66 ( 483.28 ) | 6.59 ( 5.38 ) | 2.72 ( 1.05 ) | 2.62E-104 |
| Metabolism and nutrition disorders | Decreased appetite | 84 | 6.83 ( 5.48 - 8.51 ) | 6.58 ( 395.01 ) | 6.51 ( 5.22 ) | 2.7 ( 1.03 ) | 4.10E-85 |
| Respiratory, thoracic and mediastinal disorders | Pneumothorax* | 82 | 43.35 ( 34.44 - 54.57 ) | 41.59 ( 2991.8 ) | 38.34 ( 30.46 ) | 5.26 ( 3.59 ) | 0 |
| Hepatobiliary disorders | Hepatic function abnormal | 77 | 4.25 ( 3.38 - 5.34 ) | 4.12 ( 182.18 ) | 4.09 ( 3.26 ) | 2.03 ( 0.37 ) | 2.94E-39 |
| Investigations | Hepatic enzyme increased | 46 | 34.7 ( 25.65 - 46.96 ) | 33.92 ( 1373.26 ) | 31.74 ( 23.46 ) | 4.99 ( 3.32 ) | 2.36E-292 |
| Hepatobiliary disorders | Liver disorder | 43 | 5.04 ( 3.72 - 6.83 ) | 4.95 ( 134.88 ) | 4.91 ( 3.63 ) | 2.3 ( 0.63 ) | 9.54E-29 |
| Hepatobiliary disorders | Drug-induced liver injury | 37 | 4.75 ( 3.43 - 6.59 ) | 4.68 ( 106.57 ) | 4.65 ( 3.35 ) | 2.22 ( 0.55 ) | 1.32E-22 |
| Neoplasms benign, malignant and unspecified (incl cysts and polyps) | Lung neoplasm malignant* | 27 | 14.27 ( 9.7 - 20.97 ) | 14.08 ( 319.12 ) | 13.71 ( 9.33 ) | 3.78 ( 2.11 ) | 4.81E-67 |
| Respiratory, thoracic and mediastinal disorders | Respiratory failure* | 22 | 5.91 ( 3.87 - 9.02 ) | 5.85 ( 87.62 ) | 5.79 ( 3.8 ) | 2.53 ( 0.87 ) | 3.32E-18 |
| Respiratory, thoracic and mediastinal disorders | Pulmonary hypertension* | 18 | 15.46 ( 9.65 - 24.78 ) | 15.33 ( 233.79 ) | 14.89 ( 9.29 ) | 3.9 ( 2.22 ) | 3.32E-48 |
| Renal and urinary disorders | Nephrotic syndrome | 15 | 5.58 ( 3.35 - 9.3 ) | 5.55 ( 55.35 ) | 5.5 ( 3.3 ) | 2.46 ( 0.79 ) | 3.63E-11 |
| Gastrointestinal disorders | Pneumatosis intestinalis* | 13 | 8.47 ( 4.88 - 14.68 ) | 8.42 ( 83.56 ) | 8.29 ( 4.78 ) | 3.05 ( 1.38 ) | 8.75E-17 |
| Investigations | Weight decreased | 12 | 9.14 ( 5.15 - 16.2 ) | 9.09 ( 84.81 ) | 8.94 ( 5.04 ) | 3.16 ( 1.49 ) | 6.37E-17 |
| Infections and infestations | Pneumonia bacterial* | 12 | 3.73 ( 2.11 - 6.59 ) | 3.71 ( 23.65 ) | 3.69 ( 2.09 ) | 1.88 ( 0.22 ) | 0.000172236 |
| Respiratory, thoracic and mediastinal disorders | Pulmonary alveolar haemorrhage* | 10 | 5.52 ( 2.96 - 10.31 ) | 5.5 ( 36.42 ) | 5.45 ( 2.92 ) | 2.45 ( 0.78 ) | 5.58E-07 |
| Respiratory, thoracic and mediastinal disorders | Pneumomediastinum* | 9 | 22.85 ( 11.69 - 44.67 ) | 22.75 ( 178.71 ) | 21.76 ( 11.14 ) | 4.44 ( 2.76 ) | 9.14E-35 |
| Infections and infestations | Diverticulitis* | 8 | 8.58 ( 4.26 - 17.29 ) | 8.55 ( 52.44 ) | 8.42 ( 4.18 ) | 3.07 ( 1.4 ) | 6.55E-10 |
| Respiratory, thoracic and mediastinal disorders | Pneumothorax spontaneous* | 6 | 75.67 ( 31.95 - 179.21 ) | 75.45 ( 380.67 ) | 65.29 ( 27.57 ) | 6.03 ( 4.31 ) | 9.24E-70 |
| Respiratory, thoracic and mediastinal disorders | Respiratory disorder | 6 | 7.03 ( 3.14 - 15.75 ) | 7.01 ( 30.48 ) | 6.92 ( 3.09 ) | 2.79 ( 1.12 ) | 2.38E-05 |
| Metabolism and nutrition disorders | Hypophagia | 6 | 9.18 ( 4.09 - 20.63 ) | 9.16 ( 42.81 ) | 9.01 ( 4.01 ) | 3.17 ( 1.5 ) | 1.16E-07 |
| Infections and infestations | Coronavirus infection* | 6 | 11.19 ( 4.97 - 25.16 ) | 11.16 ( 54.22 ) | 10.92 ( 4.86 ) | 3.45 ( 1.77 ) | 8.60E-10 |
| Vascular disorders | Aortic dissection | 5 | 5.55 ( 2.3 - 13.43 ) | 5.54 ( 18.41 ) | 5.49 ( 2.27 ) | 2.46 ( 0.78 ) | 0.006293237 |
| Gastrointestinal disorders | Gastric perforation | 5 | 8.49 ( 3.5 - 20.58 ) | 8.47 ( 32.39 ) | 8.34 ( 3.44 ) | 3.06 ( 1.38 ) | 1.73E-05 |
| Nervous system disorders | Taste disorder* | 5 | 7.23 ( 2.99 - 17.51 ) | 7.22 ( 26.39 ) | 7.13 ( 2.94 ) | 2.83 ( 1.16 ) | 0.000213946 |
| Cardiac disorders | Right ventricular failure* | 4 | 10.13 ( 3.76 - 27.3 ) | 10.11 ( 32.17 ) | 9.92 ( 3.68 ) | 3.31 ( 1.63 ) | 3.96E-05 |
| Gastrointestinal disorders | Faeces soft* | 4 | 19.74 ( 7.26 - 53.72 ) | 19.7 ( 68.21 ) | 18.96 ( 6.97 ) | 4.25 ( 2.55 ) | 2.86E-11 |
| Surgical and medical procedures | Lung transplant* | 3 | 717.83 ( 119.88 - 4298.43 ) | 716.74 ( 857.7 ) | 287.3 ( 47.98 ) | 8.17 ( 6.13 ) | 8.64E-130 |
| Neoplasms benign, malignant and unspecified (incl cysts and polyps) | Lung carcinoma cell type unspecified recurrent* | 3 | 44.86 ( 13.73 - 146.63 ) | 44.8 ( 117.45 ) | 41.04 ( 12.56 ) | 5.36 ( 3.62 ) | 9.74E-18 |
| Surgical and medical procedures | Lung operation* | 3 | 143.56 ( 39.48 - 522.06 ) | 143.35 ( 326.21 ) | 110.5 ( 30.39 ) | 6.79 ( 4.95 ) | 1.96E-49 |
| Surgical and medical procedures | Rehabilitation therapy* | 3 | 75.56 ( 22.34 - 255.54 ) | 75.45 ( 190.33 ) | 65.29 ( 19.31 ) | 6.03 ( 4.26 ) | 7.87E-29 |
| Gastrointestinal disorders | Inguinal hernia* | 3 | 17.09 ( 5.4 - 54.11 ) | 17.07 ( 43.81 ) | 16.51 ( 5.22 ) | 4.05 ( 2.35 ) | 1.97E-06 |

Comprehensive TTO analysis at the PT level for pirfenidone and nintedanib. Orange and blue colors indicate results for pirfenidone and nintedanib, respectively. TTO, time to onset; n, reported cases; Min, minimum; Max: maximum; IQR, interquartile range; q1, 1/4 quantile; q3, 3/4 quantile; SD, standard deviation; SE, standard error. SOC, system organ class; PT, preferred term.

| PT name | n | min | max | median | iqr | q1 | q3 | mean | sd | se |
| --- | --- | --- | --- | --- | --- | --- | --- | --- | --- | --- |
| Decreased appetite | 27 | 2 | 713 | 41 | 128.5 | 5 | 133.5 | 120.8148148 | 194.3161653 | 37.39616344 |
| Hepatic function abnormal | 19 | 2 | 693 | 61 | 81 | 31 | 112 | 110.3684211 | 157.491873 | 36.13111362 |
| Platelet count decreased | 13 | 11 | 224 | 34 | 76 | 24 | 100 | 71 | 75.28944149 | 20.88153398 |
| Pneumothorax | 12 | 14 | 489 | 61 | 146 | 20.75 | 166.75 | 126.3333333 | 150.2950633 | 43.38644763 |
| Pneumonia | 8 | 3 | 713 | 45 | 301.25 | 21.75 | 323 | 187.625 | 253.6031419 | 89.66225068 |
| Malaise | 7 | 3 | 425 | 14 | 76 | 8.5 | 84.5 | 89.71428571 | 156.2686941 | 59.06401462 |
| Photosensitivity reaction | 7 | 57 | 194 | 121 | 77.5 | 73 | 150.5 | 117 | 51.57841926 | 19.49481005 |
| White blood cell count decreased | 7 | 21 | 35 | 35 | 2 | 33 | 35 | 32.42857143 | 5.255382728 | 1.986347963 |
| Agranulocytosis | 6 | 24 | 580 | 48.5 | 52.5 | 27.5 | 80 | 135.3333333 | 219.1599112 | 89.47165908 |
| Hepatic function abnormal | 49 | 0 | 594 | 12 | 43 | 5 | 48 | 65.34693878 | 132.6719876 | 18.95314109 |
| Decreased appetite | 49 | 0 | 995 | 69 | 232 | 13 | 245 | 174.3265306 | 231.4300099 | 33.06142999 |
| Diarrhoea | 46 | 0 | 1183 | 108.5 | 231 | 25.5 | 256.5 | 186.5217391 | 254.4671766 | 37.51913829 |
| Liver disorder | 35 | 0 | 371 | 28 | 77 | 7.5 | 84.5 | 78.77142857 | 114.9498831 | 19.43007656 |
| Pneumothorax | 34 | 1 | 699 | 205.5 | 311.25 | 85.5 | 396.75 | 239.7941176 | 187.4944491 | 32.15503275 |
| Death | 24 | 13 | 818 | 181.5 | 186 | 124.5 | 310.5 | 249.875 | 203.7535409 | 41.59101738 |
| Drug-induced liver injury | 22 | 0 | 158 | 14 | 27 | 4.25 | 31.25 | 29.13636364 | 39.44410808 | 8.409512099 |
| Pneumonia | 18 | 1 | 781 | 91.5 | 237 | 32.25 | 269.25 | 189.7777778 | 241.1905655 | 56.84916148 |
| Cerebral infarction | 14 | 1 | 1324 | 146 | 246.5 | 16.75 | 263.25 | 220.5714286 | 344.6127625 | 92.10163487 |
| Platelet count decreased | 13 | 3 | 567 | 26 | 39 | 14 | 53 | 78.53846154 | 152.1937446 | 42.21095 |
| Hepatic enzyme increased | 10 | 2 | 241 | 7 | 17.75 | 3.75 | 21.5 | 36.6 | 73.94322146 | 23.38289973 |
| Vomiting | 10 | 0 | 291 | 7.5 | 53 | 1 | 54 | 50.1 | 91.80347367 | 29.03080739 |
